# *In situ* mass spectrometry imaging reveals heterogeneous glycogen stores in human normal and cancerous tissues

**DOI:** 10.15252/emmm.202216029

**Published:** 2022-09-05

**Authors:** Lyndsay E A Young, Lindsey R Conroy, Harrison A Clarke, Tara R Hawkinson, Kayli E Bolton, William C Sanders, Josephine E Chang, Madison B Webb, Warren J Alilain, Craig W Vander Kooi, Richard R Drake, Douglas A Andres, Tom C Badgett, Lars M Wagner, Derek B Allison, Ramon C Sun, Matthew S Gentry

**Affiliations:** ^1^ Department of Molecular and Cellular Biochemistry, College of Medicine University of Kentucky Lexington KY USA; ^2^ Markey Cancer Center University of Kentucky Lexington KY USA; ^3^ Department of Neuroscience, College of Medicine University of Kentucky Lexington KY USA; ^4^ Spinal Cord and Brain Injury Research Center University of Kentucky Lexington KY USA; ^5^ Cell and Molecular Pharmacology and Experimental Therapeutics Medical University of South Carolina Charleston SC USA; ^6^ Pediatric Hematology‐Oncology, College of Medicine University of Kentucky Lexington KY USA; ^7^ Pediatric Hematology‐Oncology Duke University Durham NC USA; ^8^ Department of Pathology and Laboratory Medicine, College of Medicine University of Kentucky Lexington KY USA; ^9^ Department of Biochemistry & Molecular Biology, College of Medicine University of Florida Gainesville FL USA; ^10^ Center for Advanced Spatial Biomolecule Research University of Florida Gainesville FL USA

**Keywords:** Ewing sarcoma, glycogen, glycogen storage disease, MALDI imaging, spatial metabolism, Cancer, Methods & Resources, Musculoskeletal System

## Abstract

Glycogen dysregulation is a hallmark of aging, and aberrant glycogen drives metabolic reprogramming and pathogenesis in multiple diseases. However, glycogen heterogeneity in healthy and diseased tissues remains largely unknown. Herein, we describe a method to define spatial glycogen architecture in mouse and human tissues using matrix‐assisted laser desorption/ionization mass spectrometry imaging. This assay provides robust and sensitive spatial glycogen quantification and architecture characterization in the brain, liver, kidney, testis, lung, bladder, and even the bone. Armed with this tool, we interrogated glycogen spatial distribution and architecture in different types of human cancers. We demonstrate that glycogen stores and architecture are heterogeneous among diseases. Additionally, we observe unique hyperphosphorylated glycogen accumulation in Ewing sarcoma, a pediatric bone cancer. Using preclinical models, we correct glycogen hyperphosphorylation in Ewing sarcoma through genetic and pharmacological interventions that ablate *in vivo* tumor growth, demonstrating the clinical therapeutic potential of targeting glycogen in Ewing sarcoma.

## Graphical abstract

Development of a MALDI‐based assay for the spatial quantification of microenvironmental glycogen and glycogen biochemical architecture. Hyperphosphorylated glycogen was discovered in human Ewing sarcoma. Targeting tumor‐specific glycogen may be a potential therapeutic approach for Ewing sarcoma.

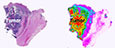

**The paper explained**

**Problem**

Physiological glycogen levels often fall below the detection limit of current histopathological methodologies. Due to the technical gap in the tools available, glycogen heterogeneity and spatial distribution in healthy and diseased tissues remains largely unknown as does the role of excess glycogen in driving pathogenesis. Development of a new method that combines glycogen architectural information with spatial distribution would be a major advancement to aid in our understanding of glycogen metabolism in both normal and disease conditions.

**Results**

Herein, we introduce a robust and sensitive workflow that provides deep interrogation of glycogen content, architecture, and spatial distribution in an array of healthy and diseased mammalian tissues, including several human cancers. Armed with this new tool, we demonstrate that glycogen levels are heterogeneous among both healthy and diseased tissues. Importantly, we identify structurally unique glycogen as a clinical feature of the pediatric cancer, Ewing sarcoma. Furthermore, we demonstrate the therapeutic potential of targeting the unique glycogen in Ewing sarcoma preclinical models.

**Impact**

Collectively, our workflow provides a sensitive and precise method to interrogate the spatial glycogen architecture and localization *in situ*. Furthermore, our data support aberrant glycogen as a clinical hallmark of Ewing sarcoma and highlight multiple therapeutic entry points for drug discovery against glycogen for the treatment of this pediatric cancer.

## Introduction

Glycogen is both an intracellular metabolite and a macromolecule with a mass that can be altered by several orders of magnitude via release of glucose‐1‐phosphate upon extracellular stimuli (Persson *et al*, 2020). Over a hundred years of glycogen‐centric research has established foundational concepts regarding: metabolism (Bernard, 1857), protein structure–function (Fischer & Krebs, 1955), and intracellular signaling (Sun *et al*, 2019; Liu *et al*, 2021). Glycogen is metabolically dynamic (Prats *et al*, 2018) and can be directly channeled to other metabolic processes including glycolysis and the Krebs cycle for ATP production (Nordlie *et al*, 1999). Administration of ^13^C‐glucose in human volunteers demonstrated glucose flux through glycogen in minutes under nonfasting conditions, suggesting that active glycogen synthesis and glycogenolysis play unknown (yet‐to‐be discovered) roles in organismal physiology (Oz *et al*, 2007). A recent study revealed glycogen as the major contributor to glycolytic intermediates in most major organs under physiological conditions (TeSlaa *et al*, 2021). In addition, glycogen supplies metabolite pools for unique cellular processes. Glycogen metabolism has recently been linked to the modulation of epigenetics via nuclear glycogenolysis to supply acetyl‐CoA (Sun *et al*, 2019), and liquid‐phase separation of protein‐bound glycogen is a driver of both liver tumorigenesis and proliferation (Liu *et al*, 2021).

Glycogen is comprised of α‐1,4‐ and α‐1,6‐linked linear glucose polymers that enable maximum packing efficiency (Roach, 2002). Glycogen biosynthesis is achieved through the stepwise actions of glycogen synthase (GYS), forming α‐1,4‐glycosidic linkages, and branching enzyme (BE), adding α‐1,6‐glycosidic linkages every 10–15 glucose residues. Additionally, phosphate is covalently attached at glucose hydroxyls during synthesis (Roach, 2015). Glucose is released from glycogen by the actions of glycogen phosphorylase (GP) and glycogen debranching enzyme (GDE; Brewer & Gentry, 2019). Glycogen architecture encompasses the modeling of α‐1,6‐branches, glucose chain length, and total phosphate esters, which are modulated by the glycogen phosphatase laforin (Worby *et al*, 2006; Adeva‐Andany *et al*, 2016). Together, these architectural parameters define the granular size, crystallinity, and solubility of a glycogen molecule within a cell. For example, glucose chain length directly impacts formation of large (50–100 μm), pathogenic glycogen aggregates also known as polyglucosan bodies (PGBs). PGB formation and phase separation render PGBs inaccessible to enzymes (Sullivan *et al*, 2017; Persson *et al*, 2020; Liu *et al*, 2021). PGBs have also been identified in the pleural perfusion of the lung (Röcken *et al*, 1996), aging prostate (Röcken *et al*, 1996), Parkinson's disease (Riba *et al*, 2019), Alzheimer's disease (Riba *et al*, 2019), and many types of cancers (Christian *et al*, 2005). However, the disease‐specific pathological roles of these glycogen‐like aggregates and differences in their architecture remain to be defined. Purified glycogen can be assessed using chromatography‐based methods to quantify glucose chain length and phosphate levels, but these methods lack sensitivity, tissue‐specific spatial resolution, and structural information (Young *et al*, 2020). Of note, periodic acid‐Schiff (PAS) is used to assess spatial glycogen; however, due to low specificity and sensitivity, its application is limited to the liver, muscle, and certain types of high glycogen cancers (Aterman & Norkin, 1963). Glycogen stores and architecture can change dramatically in response to stimuli or microenvironmental changes during exercise and disease. Additionally, glycogen levels vary dramatically among cell types and subtissue regions. Therefore, a new method that combines architectural information with spatial distribution and the sensitivity of mass spectrometry would be a major advancement to aid in our understanding of glycogen metabolism in both physiological and disease conditions.

Matrix‐assisted laser desorption/ionization mass spectrometry imaging (MALDI‐MSI) is at the forefront of technological innovations while being implemented to make significant clinical and biological discoveries in the last decade (Powers *et al*, 2015). Enzyme‐assisted MALDI‐MSI is a relatively new technique that can release a quantifiable metabolic product, enabling the utilization of the vast amount of clinical resources stored in the form of formalin‐fixed paraffin‐embedded (FFPE) tissues (Clift *et al*, 2021; Conroy *et al*, 2021). We previously demonstrated the early application and potential of MALDI‐MSI to visually quantify glycogen *in situ* (Hawkinson & Sun, 2022). Herein, we introduce a comprehensive workflow using ion‐mobility MALDI‐MSI for unambiguous and deep interrogation of glycogen levels, architecture, and spatial origin in mammalian tissues. Armed with this new tool, we report architecturally unique glycogen localization in an array of mouse and human healthy and diseased tissues. Most interestingly, we identified excess PGBs in the pediatric cancer, Ewing sarcoma. Furthermore, we demonstrate the therapeutic potential of PGBs in Ewing sarcoma using two different modalities targeting PGBs that ablated xenograft tumor growth *in vivo*.

## Results

### Enzyme‐assisted MALDI imaging of spatial glycogen *in situ*

**Figure 1:**
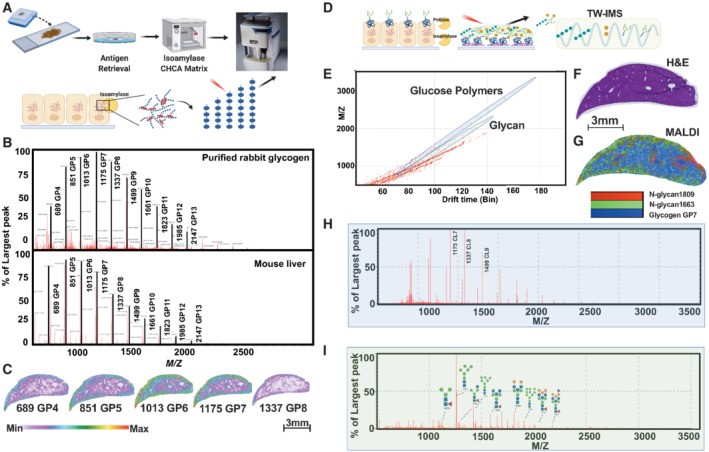
*In situ* digestion of glycogen by isoamylase releases glucose polymers, detectable by MALDI‐MSI A(*Top*) Schematic of MALDI‐MSI workflow: FFPE tissue slices (4 μm) processed through antigen retrieval, enzyme digestion, and matrix application followed by ionization by argon laser and detection by time‐of‐flight (TOF) detector. (*Bottom*) Schematic of isoamylase digestion of glycogen from tissue sections cleaving alpha‐1,6‐glycosidic bonds, releasing linear glucose polymers.BRepresentative ion chromatogram of linear glucose polymers (GP) detected by MALDI‐TOF following isoamylase digestion of (*top*) purified rabbit liver glycogen and (*bottom*) mouse liver tissue. GP4 to GP13 and their masses (rounded to the nearest one) were highlighted for better visualization.CSpatial distribution of unique GP4‐8 from a wild‐type (WT) mouse liver. The image displays a heatmap with gradient of intensity from white (least abundant) to red (most abundant). Scale bar is represented below the images.DSchematic of a multiplex analysis of glucose polymers and N‐linked glycans after PNGase F and isoamylase digestion followed by traveling wave ion mobility separation (TW‐IMS).EScatter plot of monoisotopic mass versus drift time in the ion mobility cell for glucose polymers and N‐glycans from mouse liver tissue.FHematoxylin and eosin (H&E)‐stained cross section of a mouse liver. Scale bar is representative of both (F) and (G). Image is also used in Fig 2A.GOverlay MALDI‐IMS image of glycogen (CL7, *m/z* = 1,175 blue) and N‐linked glycans (*m/z* = 1,663 green; *m/z* = 1,809 red) of an immediate adjacent slice to the H&E section of (F).HRelative abundance of *m/z* extracted from the glucose polymer regions of (E) representing glycogen chain lengths ranging from *m/z* = 500–3,500.IRelative abundance of *m/z* extracted from the glycan regions of (E) representing N‐linked glycans from *m/z* = 500–3,500.

MALDI imaging has been employed for the spatial profiling of N‐linked glycans from FFPE tissues after enzymatic hydrolysis of N‐linked glycans from proteins using peptide‐N‐glycosidase F (PNGase F) with spatial details ranging from macro‐to micro‐tissue structures (Drake *et al*, 2018; Stanback *et al*, 2021). We hypothesized that a similar approach could be adapted for the spatial profiling of glycogen in tissues. For the enzymatic digestion of glycogen, we employed isoamylase (Glycogen 6‐glucanohydrolase, Megazyme), a bacterial enzyme that specifically cleaves the glycogen α‐1,6‐glycosidic bonds to release linear glucose polymers from glycogen that range 3–25 glucose units in length (Harada *et al*, 1972; Fig 1A). To test the utility of isoamylase in MALDI‐MSI, purified rabbit liver glycogen was directly spotted and dried on a microscope slide, processed through an antigen retrieval step, and then isoamylase (3 U) was applied using a high‐velocity dry sprayer to cleave glycogen into glucose polymers. Finally, α‐cyano‐4‐hydroxycinnamic acid (CHCA) ionization matrix was applied using the same dry sprayer with modified parameters (Fig 1A). Released glucose polymers were analyzed by MALDI mass spectrometry using a Waters Synapt G2 XS ion mobility‐enabled mass spectrometer equipped with an Nd:YAG laser. Two‐hour isoamylase digestion generated stepwise peaks that are 162 *m/z* apart as recorded by the time‐of‐flight (TOF) mass detector (Fig 1B). 162 *m/z* corresponds to the 1 glucose unit difference among polymers and agrees with previously published glucose polymer patterns established by high‐performance anion‐exchange chromatography coupled with pulsed amperometric detection (HPAC‐PAD; Hanashiro *et al*, 1996).

Glycogen phosphorylation is a critical biochemical modification regulated by the glycogen phosphatase laforin (Worby *et al*, 2006; Tagliabracci *et al*, 2007). Phosphate can be covalently linked to glycogen at the C2‐, C3‐, and C6‐hydroxyl residues of glucose (Appendix Fig S1A; Tagliabracci *et al*, 2011). Glycogen hyperphosphorylation results in PGBs that impact glycogen turnover, and perturbations in this process have deleterious consequences as mutations in the gene encoding laforin result in the fatal childhood dementia and progressive myoclonus epilepsy Lafora disease (Turnbull *et al*, 2016; Gentry *et al*, 2018). Phosphorylated glycogen is difficult to quantify using conventional biochemical methods. In addition to glucose polymers, we were also able to identify phosphorylated glucose polymers (Appendix Fig S1B). Thus, we can characterize multiple parameters of glycogen architecture, that is, chain length and phosphate levels, using MALDI mass spectrometry.

To test whether this method can be applied to whole tissue, we processed FFPE C57BL/6J mouse liver through the workflow. Liver is the most well‐known site of glycogen storage and plays a key role in regulating whole‐body blood glucose concentration (Hultman & Nilsson, 1971). FFPE 3‐month‐old mouse liver was sectioned at 4 μm thickness followed by sequential application of isoamylase and CHCA by high‐velocity dry spraying and analyzed by MALDI‐MSI. Mouse liver exhibited a similar glucose polymer distribution pattern compared with purified glycogen (Fig 1B). The glycogen spatial distribution within the liver was generated using the relative abundance of the most prominent glucose polymers, which are 4–8 glucose units in length (Fig 1C). To test whether the workflow is robust across different MALDI‐MSI platforms, an adjacent section of liver tissue was scanned for glucose polymer distribution using a Bruker TIMS‐TOF Flex instrument after parallel treatment by isoamylase and application of CHCA at the Medical University of South Carolina. We observed nearly identical chain length distribution pattern and glycogen regional distribution between two different platforms (Appendix Fig S1C and D). These data confirm that the method is robust and reproducible across institutions and different mass spectrometer platforms.

Traveling wave ion mobility separation is a relatively new technology that provides *de novo* separation of molecular ions with similar *m/z* but different collision cross section (Shvartsburg & Smith, 2008). We hypothesized that N‐linked glycans and glycogen could be multiplexed in one assay with the aid of ion mobility separation. To test this hypothesis, we performed co‐spraying of PNGase F and isoamylase (Fig 1D) and incubated the slide for 2 h followed by CHCA matrix application and MALDI‐MSI analysis. As predicted, co‐treatment of PNGase F and isoamylase produced both N‐linked glycans and glycogen‐derived glucose polymers (Fig 1E–G and Appendix Fig S1E–G) that displayed differential migration through the traveling wave ion mobility chamber (Fig 1E, H and I, and Appendix Fig S1E–G). Multiplexed imaging of both N‐linked glycans and glycogen revealed distinct spatial differences through the cross section of WT mouse liver (Fig 1G). Thus, this MALDI‐MSI multiplexed workflow provides quantification of both N‐linked glycans and glycogen while also providing spatial distribution throughout the tissue.

### Heterogeneous glycogen spatial distribution in major organs of C57BL/6J mice

**Figure 2:**
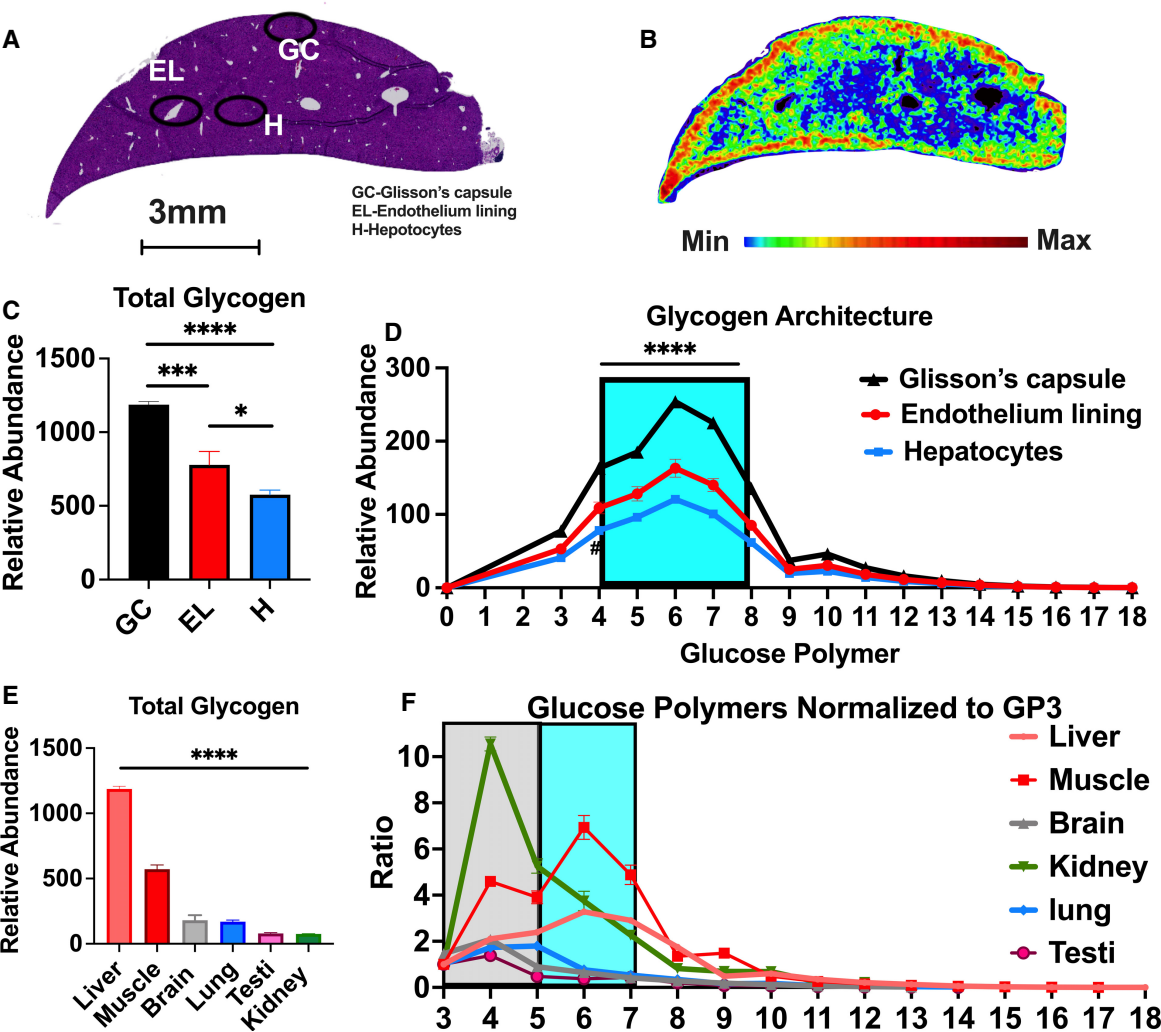
Spatial analysis of glycogen structure in a mouse liver AHematoxylin and eosin (H&E)‐stained cross section of a mouse liver. Three separate regions were extracted for regional structural analysis. Glisson's capsule (GC), endothelium lining of the central vein (EL), and hepatocyte nodule (H). Scale bar is representative of both (A) and (B). Image is also used in Fig 1F.BSpatial distribution showing regional and relative abundance of glucose polymer (GP) 7 (*m/z* = 1,175) of the immediate adjacent slice from the mouse liver in (A). The image displays a heatmap gradient of intensity with blue (least abundant) to red (most abundant).CTotal glycogen calculated from the sum of CL3‐CL18 from the different anatomical regions in (A). Values are presented as mean ± standard error (*n* = 3 technical replicates per region). *0.01 < *P* < 0.05; ***P < 0.001; ****P < 0.0001, analyzed by one‐way ANOVA with Tukey's multiple comparison for total glycogen.DGlycogen structure defined by distribution of released glucose polymers as a representation of glycogen chain length (CL) distribution from ROIs defined in (A) with CL4‐8 highlighted in cyan demonstrating differences between most abundant CL among ROIs. Values are presented as mean ± standard error (*n* = 3 technical replicates per region). ****P < 0.0001, analyzed by two‐way ANOVA with Tukey's multiple comparisons for each glycogen chain length.EQuantification of relative abundance of total glycogen calculated from the sum of CL3‐CL18 in mouse liver, skeletal muscle, brain, lung, testes, and kidney tissues. Values are presented as mean ± standard error (*n* = 3 technical replicates per region). ****P < 0.0001, analyzed by one‐way ANOVA with Tukey's multiple comparison for total glycogen.FGlycogen structure represented by normalized chain length distribution (ration to CL3) of each mouse tissue from (E) with CL3‐CL5 highlighted in gray and CL5‐CL6 highlighted in cyan demonstrating differences between most abundant CL among mouse tissues. Values are presented as mean ± standard error (*n* = 3 technical replicates per region).

Glycogen has been reported in multiple tissues in both mice and humans (Zois *et al*, 2014; Adeva‐Andany *et al*, 2016). However, a detailed simultaneous spatial distribution and architectural assessment remain a critical knowledge gap in understanding the biological roles of glycogen in these tissues. We applied the MALDI‐MSI method to examine glycogen composition in multiple wild‐type C57BL/6J mouse tissues. First, we performed spatial glycogen analysis of the mouse liver. Heatmap distribution of the most abundant glucose polymer ion, DP 7, exhibits heterogeneous localization of glycogen in the mouse liver. Strikingly, the connective tissue layer encapsulating the liver known as the Glisson's capsule (GC) and the endothelium lining (EL) of the central vein have significantly higher levels of glycogen compared with hepatocytes (H) (Fig 2A–C). Furthermore, there was a gradient of glycogen stores radiating away from the central vein (EL) as evidenced by pixel analyses from three separate regions of the tissue cross section (Appendix Fig S2A). The relative abundance of each glucose polymer recorded by MALDI‐MSI also contains data regarding glycogen chain length, a crucial architectural parameter of glycogen. MALDI‐MSI analysis of glycogen chain length suggested a major decrease in glycogen occurs between chain length 4 and 8 between the histological regions (Fig 2D), while no significant decrease between chain length 9 and 18 among different anatomical regions of the mouse liver.

To define glycogen spatial distribution and architecture in other mouse organs, we performed MALDI‐MSI analysis of glycogen using mouse kidney, testis, lung, muscle, and bladder (Appendix Fig S2B–D). In agreement with previously published results, liver and muscle are major sites of glycogen accumulation (Fig 2E). Kidney, testis, lung, and bladder all display unique, heterogeneous glycogen spatial distribution patterns and unique chain length distribution (Fig 2F and Appendix Fig S2A–C). For example, each tissue exhibited glycogen accumulation in the outer fibroconnective tissue layer of the kidney, testis, and the lung, suggesting a structural role for glycogen within these tissues (Appendix Fig S2B–D). Of note, the primary site of glycogen accumulation in the bladder is the urothelial lining layer and not the outer smooth muscle capsule (Appendix Fig S2D). Mouse organs exhibited diverse chain length distribution with kidney, lung, and brain displaying predominantly shorter chain length compared with muscle and liver glycogen (Fig 2F). Collectively, our data suggest tissue‐specific regulation of glycogen metabolism, and diverse metabolic utilization for glycogen among different mouse organs and tissue regions. These data will be valuable resources for researchers working on glycogen in an array of tissues.

### MALDI‐MSI analysis reveals distinct tissue glycogen distribution in human samples

**Figure 3:**
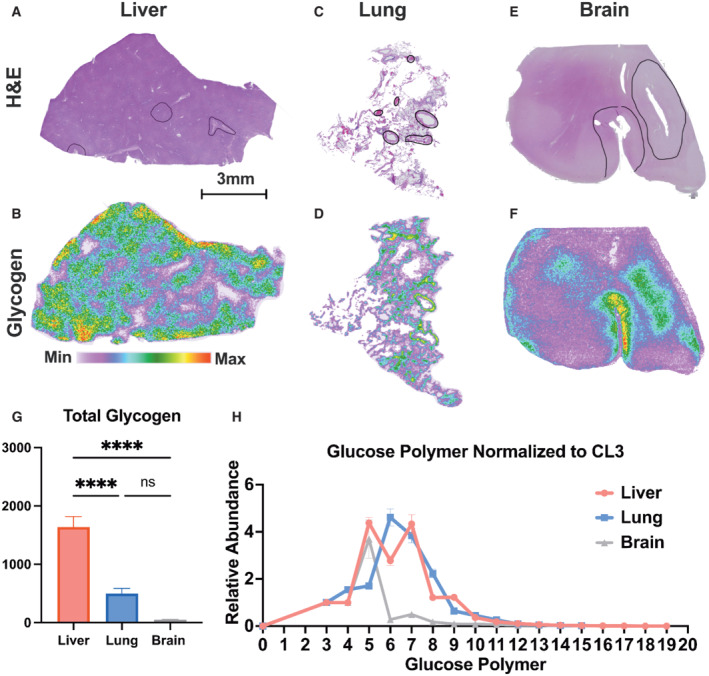
Spatial analysis of human liver, lung, and brain glycogen structure AHematoxylin and eosin (H&E)‐stained cross section of a human liver slice. Scale bar is representative of (A–F). Image is also used in Appendix Fig S3A.BSpatial distribution and relative abundance of glycogen (represented by CL7) in an immediate adjacent tissue slice from (A). The image displays a heatmap gradient of intensity from white (least abundant) to red (most abundant) which is representative of (B), (D), and (F).CH&E‐stained cross section of a human lung slice. Image is also used in Appendix Fig S3D.DSpatial distribution and relative abundance of glycogen (represented by CL7) in an immediate adjacent tissue slice from (C).EH&E‐stained cross section of frontal cortical region of a human brain tissue. Image is also used in Appendix Fig S3G.FSpatial distribution and relative abundance of glycogen (represented by CL7) in an immediate adjacent tissue slice from (E).GQuantification of relative abundance of total glycogen calculated from the sum of CL3‐CL18 in human liver, lung, and brain. Values are presented as mean ± standard error (*n* = 3 technical replicates per region). ****P < 0.0001, analyzed by one‐way ANOVA with Tukey's multiple comparison for total glycogen.HGlycogen structure represented by normalized chain length distribution (ratio to CL3) of each human liver, lung and brain from (G). Values are presented as mean ± standard error (*n* = 3 technical replicates per region).

To assess glycogen distribution in normal human tissues, we histologically assessed over 300 tissue slides collected during surgery and identified human liver, lung, and brain samples that lacked any identifiable pathological features. In the human liver, glycogen was observed in the hepatocyte lobules where glycogen storage has previously been reported in the hepatocytes (Fig 3A; Bollen *et al*, 1998). Interestingly, cellular layers that comprise the portal veins contain very low levels of glycogen (Fig 3A and B). Molecular analysis of glycogen chain length revealed similarities and differences between human liver glycogen and mouse liver glycogen. Similar to mice, major differences in glycogen stores were observed between different regions and the difference is driven by changes in chain length 4–8 with no significant differences in chain length ≥ 10 (Appendix Fig S3A and B). In addition to human liver, we also performed MALDI‐MSI on human lung (Fig 3C and D, and Appendix Fig S3D and E) and the frontal cortex from an aged human brain (Fig 3E and F, and Appendix Fig S3G and H). Similar to mice, MALDI‐MSI analysis defined heterogeneous distribution of glycogen in unique regions of both the human lung and the brain (Fig 3B, D and F). Glycogen is present throughout the lung including cellular layers surrounding the alveoli sac (Fig 3D and Appendix Fig S3D and E). In the aged brain, gray matter tracks display higher glycogen concentrations compared with white matter tracks (Fig 3F and Appendix Fig S3G and H). To further define human glycogen architecture, we analyzed phosphorylated chain length distribution of human liver, lung, and brain. Phosphorylated chain length displayed similar polymeric distribution compared with the nonphosphorylated glycogen with unique differences among the highest phosphorylated glycogen chain length. For example, in lung and liver, phosphorylated polymers were highest at a chain length of 3 and 4, respectively, while unphosphorylated chains displayed highest abundance at lengths of 5–7 for liver, and 6–8 for lung (Appendix Fig S3C, F and I). Our analysis demonstrates that shorter chain lengths in both liver and lung tissues exhibit the highest phosphate content (Appendix Fig S3C, F and I). Cumulatively, these data suggest strategic phosphate placement within the glycogen molecule (Tagliabracci *et al*, 2007; Irimia *et al*, 2015).

### Tumor‐specific glycogen stores and spatial distribution

**Figure 4:**
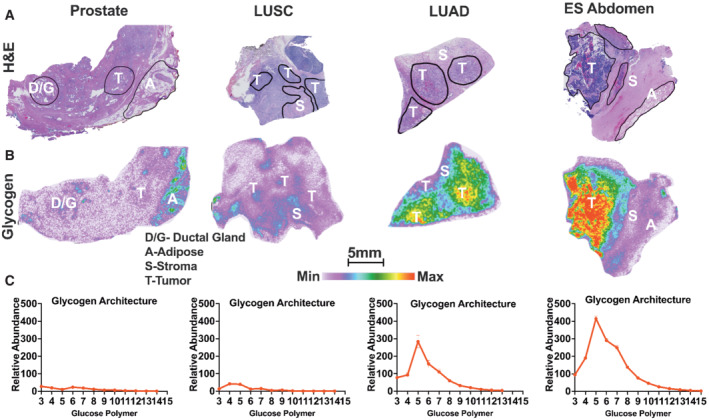
Spatial glycogen abundance across human prostate cancer, nonsmall‐cell lung cancers, and Ewing sarcoma tissues AHematoxylin and eosin (H&E)‐stained cross section of adenocarcinoma of the prostate, lung squamous cell carcinoma (LUSC), lung adenocarcinoma (LUAD), and Ewing sarcoma (ES) of the abdomen tumor. Annotated regions are T‐tumor, S‐stroma, D/G‐duct/glandular, and A‐adipose tissue. Scale bar is representative of (A) and (B). Images are also used in Fig 5D and Appendix Figs S4A and S6G.BSpatial distribution and relative abundance of glycogen (represented by CL7) from an immediate adjacent resected tissue section shown in (A). The image displays a heatmap gradient of intensity with white (least abundant) to red (most abundant). Images are also used in Fig 5D and Appendix Figs S4E and S6G.CGlycogen structure defined by distribution of released glucose polymers as representation of glycogen chain length (CL) distribution from tumor regions shown in (A). Values are presented as mean ± standard error (*n* = 3 technical replicates per region).

Increased glycogen storage has been reported in multiple cancer types including breast, lung, bladder, renal, pancreatic, and liver from pooled tissues (Rousset *et al*, 1981; Ritterson Lew *et al*, 2015; Sun *et al*, 2019; Wolfe *et al*, 2021; Xie *et al*, 2021). However, little is known about glycogen architecture and spatial distribution within the tumor microenvironment of different cancer subtypes. Using the MALDI‐MSI method, we compared glycogen architecture of prostate, non‐small cell lung cancer (NSCLC), and Ewing sarcoma (ES) tumors (Fig 4). The extent of glycogen accumulation is strikingly diverse across tumor types. In low‐grade prostate tumor tissue, glycogen is largely absent within the tumor, but it is visible in glandular ducts known as corpora amylacea, and adjacent adipose tissue, visible by hematoxylin and eosin (H&E) staining (Fig 4A–C and Appendix Fig S4). Lung adenocarcinoma (LUAD) and squamous cell carcinoma (LUSC) are two of the most common histological subtypes of NSCLC. Significant disparities exist between LUAD and LUSC from confounding factors such as geographic regions, mutation status, and smoking frequency (Kawase *et al*, 2011). As expected, intratumoral glycogen was observed in LUAD and not in LUSC (Fig 4A–C). Interestingly, glycogen was identified in stroma, blood vessel walls, and residual cartilage in LUSC (Fig 4A and B). Collectively, these results highlight the need for spatial glycogen analysis as pooled LUSC and prostate samples would likely result in a false positive for intratumoral glycogen accumulation based on glycogen in nontumor regions. Strikingly, an abdominal ES tumor exhibited the highest amount of glycogen of the different cancer types (Fig 4A–C). ES contains twofold higher glycogen than LUAD and eightfold higher glycogen than LUSC. Given the gradient of glycogen accumulation between different tumor subtypes, we hypothesize that glycogen is not a universal feature of tumorigenesis but could be a unique opportunity for targeted intervention.

### Targeting glycogen excess phenotype in ES halts tumor growth *in vivo*

**Figure 5:**
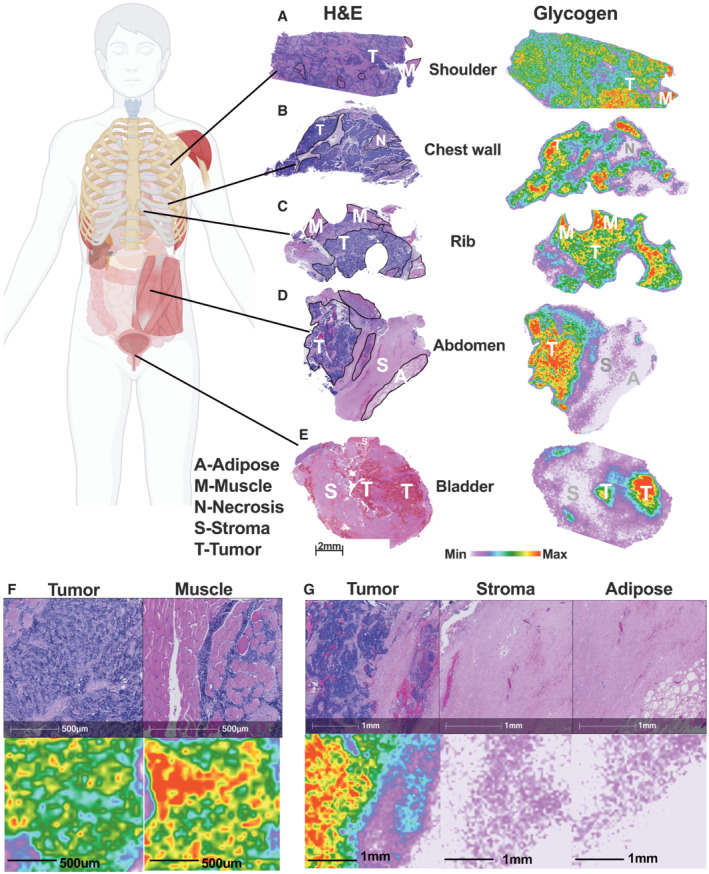
Five Ewing sarcoma tumor tissues with intratumoral microenvironmental glycogen A(*Left*) Hematoxylin and eosin (H&E)‐stained cross section of an Ewing sarcoma (ES) of the shoulder tumor. Annotated regions are annotated as T‐tumor and M‐muscle. (*Right*) Spatial distribution and relative abundance of glycogen (represented by CL7) from an immediate adjacent resected tissue section. The image displays a heatmap gradient of intensity from white (least abundant) to red (most abundant). Scale bar and intensity gradient below are representative of all images in (A–E). Images are also used in Appendix Figs S5C and S6D.B(*Left*) H&E‐stained cross section of an ES of the chest wall tumor. Annotated regions are annotated as T‐tumor and N‐necrosis. (*Right*) Spatial distribution and relative abundance of glycogen (represented by CL7) from an immediate adjacent resected tissue section. Images are also used in Appendix Figs S5E and S6E.C(*Left*) H&E‐stained cross section of an ES of the rib tumor. Annotated regions are annotated as T‐tumor and N‐necrosis. (*Right*) Spatial distribution and relative abundance of glycogen (represented by CL7) from an immediate adjacent resected tissue section. Images are also used in Appendix Fig S6F.D(*Left*) H&E‐stained cross section of an ES of the abdomen tumor. Annotated regions are T‐tumor, S‐stroma, and A‐adipose tissue. (*Right*) Spatial distribution and relative abundance of glycogen (represented by CL7) from an immediate adjacent resected tissue section. Images are also used in Fig 4A and Appendix Fig S6G.E(*Left*) H&E‐stained cross section of an ES of the bladder tumor. Annotated regions are T‐tumor and S‐stroma. (*Right*) Spatial distribution and relative abundance of glycogen (represented by CL7) from an immediate adjacent resected tissue section. Images are also used in Appendix Fig S6H.FMagnified H&E cross section of (*top*, *left*) tumor, and (*top*, *right*) muscle tissue from ES rib with magnified image of CL7 (*m/z* = 1,175) of (*bottom*, *left*) tumor, and (*bottom*, *right*) muscle tissues.GMagnified H&E cross section of (*top*, *left*) tumor, (*top*, *middle*) stroma, and (*top*, *right*) adipose tissues from ES abdomen with magnified image of CL7 (*m/z* = 1,175) of (*bottom*, *left*) tumor, (*bottom*, *middle*) stroma and (*bottom*, *right*) adipose tissues.

**Figure 6:**
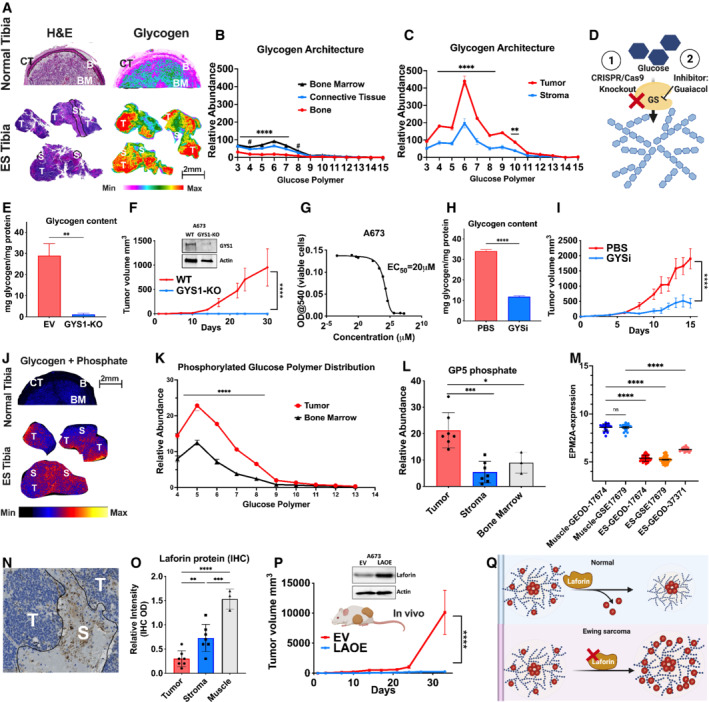
Glycogen accumulation is a therapeutic target in preclinical models of Ewing sarcoma A(*Left*) Hematoxylin and eosin (H&E) stained cross section of a normal human tibia (*Top*) resected at the same time as the Ewing sarcoma (ES) of the tibia tumor (*Bottom*). Annotated regions are CT‐connective tissue, B‐decalcified bone, BM‐bone marrow, T‐tumor, and S‐stroma. (*Right*) Spatial distribution and relative abundance of glycogen (represented by CL7) from an immediate adjacent resected tissue section shown in (A). The image displays a heatmap gradient of intensity with white (least abundant) to red (most abundant). Scale bar and intensity gradient below are representative of all images in (A).BGlycogen structure defined by distribution of released glucose polymers as representation of glycogen chain length (CL) distribution from normal tibia in (A). Values are presented as mean ± standard error (*n* = 3 technical replicates per region). ^#^0.01 < *P* < 0.05; ****P < 0.0001, analyzed by two‐way ANOVA with Tukey's multiple comparison for each glycogen chain length.CGlycogen structure defined by distribution of released glucose polymers as representation of glycogen chain length (CL) distribution from the ES of the tibia tumor in (A). Values are presented as mean ± standard error (*n* = 3 technical replicates per region). **0.001 < *P* < 0.01; ****P < 0.0001, analyzed by two‐tailed *t*‐test for each glycogen chain length.DSchematic of targeting glycogen synthesis by CRISPR/Cas9 knockout or small molecule inhibitor, guaiacol, to prevent glycogen synthesis.EGCMS quantitation of glycogen abundance in A673 empty vector (EV) or glycogen synthase knockout (GYS1‐KO) cells *in vitro*. Values are presented as mean ± standard error (*n* = 3 biological replicates per group). **0.001 < *P* < 0.01, analyzed by two‐tailed *t*‐test.F
*In vivo* tumor growth of A673 EV or GYS1‐KO injected in the flanks of athymic nude mice. Values are presented as mean ± standard error (*n* = 8 biological replicates per group). ****P < 0.0001, analyzed by two‐tailed *t*‐test.GHalf maximal effective concentration (EC_50_) of the small molecular inhibitor guaiacol in A673 cells *in vitro*.HGCMS quantitation of glycogen abundance in A673 WT cells treated with PBS or Guaiacol (GYSi) (20 μM). Values are presented as mean ± standard error (*n* = 3 biological replicates per group). ****P < 0.0001, analyzed by two‐tailed *t*‐test.I
*In vivo* tumor growth of A673 cells injected in the flanks of athymic nude mice. Mice received daily injection of either PBS or GYSi (30 mg/kg). Values are presented as mean ± standard error (*n* = 8 biological replicates per group). ****P < 0.0001, analyzed by two‐tailed *t*‐test.JSpatial distribution of phosphorylated CL5 of human tibia (top) and tibial ES tumor (bottom). The image displays a heatmap gradient of intensity with black (least abundant) to yellow (most abundant).KPhosphorylated chain length distribution in normal human tibia and tibial ES tumor in (J). Values are presented as mean ± standard error (*n* = 3 technical replicates per region). ****P < 0.0001, analyzed by two‐tailed *t*‐test for each glycogen chain length.LExtracted phosphorylated CL5 abundance between normal human tibia, tibial ES tumor, and stroma. Values are presented as mean ± standard error (*n* = 3 technical replicates per region). *0.01 < *P* < 0.05; ***P < 0.001, analyzed by one‐way ANOVA with Tukey's multiple comparison.MRelative mRNA abundance of *Epm2a* between normal muscle [GEOD‐17674 (*n* = 18) and GSE17679 (*n* = 18) and Ewing Sarcoma tumors [GEOD‐17674 (*n* = 44), GSE17679 (*n* = 65), and GEOD‐37371 (*n* = 20)] based on published RNAseq datasets. Values are presented as relative expression ± standard error. ****P < 0.0001, analyzed by one‐way ANOVA with Tukey's multiple comparison.NRepresentatives image of immunohistochemical staining of laforin in ES patient samples with annotation between tumor (T) and stroma (S) tissues. Tissues were scanned digitally using the Axio Scan.Z1 side scanner. Scale bar is shown below the image.OQuantification of immunohistochemical laforin staining between normal tissue (*n* = 3 technical replicates per region), tibial ES tumor (*n* = 7 technical replicates per region), and stroma of five ES tissues (*n* = 7). Values are presented as mean ± standard error. **0.001 < *P* < 0.0; ***P < 0.001; ****P < 0.0001, analyzed by one‐way ANOVA with Tukey's multiple comparison.P(*Top*) Western blot showing confirmation of laforin overexpression in A673 xenograft tumors. (*Bottom*) Tumor growth of xenografts of A673 empty vector (EV) and laforin overexpression (LAOE). Values are presented as mean ± standard error (*n* = 8 biological replicates per group). ****P < 0.0001, analyzed by two‐tailed *t*‐test.QSchematic of the role of laforin as a glycogen phosphatase. Loss of laforin leads to hyperphosphorylated glycogen in ES (*bottom*) while normal laforin function regulates glycogen phosphate content (*top*).

Ewing sarcoma tumors contained the highest glycogen accumulation in our initial screening. Therefore, we proceeded to test whether glycogen is critical for ES tumor proliferation. ES is a pediatric bone and soft tissue malignancy affecting 2.93 children per 1,000,000. Approximately 250 children and adolescents are diagnosed each year in the United States (Grunewald *et al*, 2018; American Cancer Society, 2020). The standard of care for ES patients has not significantly changed in the past few decades, and the overall survival for metastatic disease remains low. PAS+ diagnosis is a clinical feature of ES tumors, presumably high in glycogen, yet glycogen has not been evaluated as a potential therapeutic option. Utilizing our workflow, we assessed spatial glycogen architecture from a cohort of ES patient tumors resected from the shoulder, chest wall, rib, abdomen, and bladder and a primary ES tibial tumor with matched normal bone (decalcified on slide) (Figs 5A–E and 6A). Glycogen levels were dramatically higher in the intratumoral regions for all ES samples compared with surrounding tissues, including tumor‐associated stroma, adipose, and necrotic (Fig 5F and G, and Appendix Fig S5A–I). Cell type of origin for ES remains speculative; however, evidence strongly suggests ES arises from connective tissues or bone marrow mesenchymal stem cells (MSC) near the bone (Lin *et al*, 2011). Interestingly, ES glycogen abundance and glycogen phosphate were comparable to both adjacent skeletal muscle (Appendix Figs S5G–I and S6D–I) and bone marrow‐derived MSC (*in vitro* compared with the A673 model ES cell line) (Appendix Fig S5J and K). In addition, ES glycogen from tumors throughout the body was hyperbranched with significant increases in major chain lengths which suggest increased biosynthesis (Appendix Fig S5A, B and G–I).

Next, we focused our spatial and architectural analyses of ES glycogen to ES tumor and tibia from the same patient. In the normal bone tissue, glycogen predominantly accumulated in the bone marrow with less extent in the outer bone region which has been decalcified postsectioning (Fig 6A). ES tibial tumor tissue exhibited profound glycogen accumulation in the tumor in stark contrast to adjacent stroma regions or the matched normal bone (Fig 6B and C). Given the dramatically high levels of glycogen in ES, glycogen synthase (GS) was assessed as an ES therapeutic target by employing two independent modalities (Fig 6D). First, we performed CRISPR‐Cas9 knockout of glycogen synthase (*GYS1*) in A673 ES cells. *GYS1* deficiency (GYS1‐KO or −/−) dramatically decreased glycogen in A673 cells cultured as a monolayer (Fig 6E). While GYS1‐KO did not affect cell viability *in vitro* (Appendix Fig S6A), we postulate that this is due to the artificial environment of cell culture (21% O2, 25 mM glucose) with *in vitro* conditions not replicating the ES tumor microenvironment *in vivo*. Next, to test whether glycogen metabolism is required for tumor growth *in vivo*, A673 WT and GYS1‐KO cells were injected subcutaneously into athymic nude mice. Strikingly, GYS1‐KO ablated the ability for the A673 cell line to form tumors *in vivo* (Fig 6F). Furthermore, we tested whether the GYS1 small molecule inhibitor guaiacol (GYSi) could delay tumor growth *in vivo*. Guaiacol is a plant‐derived component that has been shown to achieve robust biodistribution in humans (Kuge *et al*, 2003; Kakhlon *et al*, 2018). Additionally, guaiacol was recently shown to reduce glycogen levels in a mouse model of a glycogen storage disease (GSD) without adverse effects to other organs (Kakhlon *et al*, 2018). To test efficacy of guaiacol to reduce glycogen in ES, A673 cells were grown to 80% confluency in 25 mM DMEM cell culture media. Increasing concentrations of guaiacol were added, and cells were harvested after 24 h. Guaiacol treatment resulted in a threefold reduction in total glycogen and an EC_50_ of ~20 μM (Fig 6G and H). Next, A673 WT cells were injected into athymic nude mice as subcutaneous xenografts. Daily injections of 30 mg/kg guaiacol began following tumor formation of 0.1 mm^3^, and tumor size was quantified over time. Guaiacol treatment resulted in a significant reduction in tumor growth with a threefold reduction at the experimental endpoint (Fig 6I). We then performed histochemical analyses of cleaved caspase 3 (CC3, apoptosis) and Ki‐67 (mitotic index) in tumors from both PBS (vehicle) and guaiacol‐treated cohorts of mice. We observed a mild increase in CC3 (*P* = 0.054) and a significant decrease in Ki‐67 in guaiacol‐treated ES tumors (Appendix Fig S6B and C). The difference between GYS1‐KO and guaiacol‐treated tumors is likely due to residual GYS activity and glycogen stores in the guaiacol cohort. Collectively, both genetic and pharmacological approaches suggest targeting GYS1 is a viable strategy for the treatment of ES.

Glycogen phosphorylation plays a pivotal role in glycogen architecture, metabolism, and access to degradation enzymes. Compared with normal tissues, ES glycogen exhibits marked increases in total phosphate content, reminiscent of PGBs in GSDs (Fig 6J and Appendix Fig S6D–I; Chen & Weinstein, 2016). MALDI‐MSI detected increased phosphate levels in most ES glucose polymers compared with stroma, normal muscle region, and the bone (Fig 6K and L). Glycogen hyperphosphorylation is indicative of loss of laforin activity, a glycogen phosphatase recently demonstrated to play a key role in liver cancer tumorigenesis (Turnbull *et al*, 2011, 2014; Pederson *et al*, 2013; Gentry *et al*, 2018). Based on the ES glycogen hyperphosphorylation, we hypothesized that ES tissues would display decreased laforin levels and performed a retrospective mRNA analysis in three different publicly available ES datasets, we observed significant reductions in laforin mRNA levels in three independent studies (Fig 6M). We confirmed that this genotype is translated to phenotype by performing laforin protein analysis with immunohistochemical staining in adjacent slices. We observed significantly lower laforin protein expression compared with adjacent tumor‐associated stroma and normal tissue (Fig 6N and O and Appendix Fig S6J). To test whether increased glycogen phosphorylation promotes ES tumor growth, we ectopically expressed either laforin (laforin‐OE) or empty vector (EV) in A673 and TC32 ES cells, injected cells as subcutaneous xenografts in nude mice, and quantified tumor volume over time. The overexpression of laforin in both ES cell lines dramatically inhibited tumor growth compared with control cells (Fig 6P and Appendix Fig S6K). These data support the model wherein laforin protein expression is decreased in ES tissue, resulting in increased glycogen levels and glycogen phosphorylation (Fig 6Q). Thus, reducing glycogen hyperphosphorylation is antiproliferative in ES. These data support a model whereby ES tissue has decreased expression of laforin that leads to hyperphosphorylated and excess accumulation of glycogen. Collectively, our data reveal glycogen as a promising target for ES with multiple possible therapeutic entry points for drug discovery.

## Discussion

While monosaccharides are considered simple sugars, multiple recent studies have highlighted that they are utilized to power exceedingly complex metabolic pathways. A recent paper reported that nearly 25% of brain glycogen consists of glucosamine, a key building block for N‐linked glycosylation, and glycogen‐associated glucosamine represents a substantial substrate pool for the biosynthesis of N‐linked glycans (Sun *et al*, 2021). Furthermore, additional studies identified novel roles of nuclear glycogenolysis as a supply of acetyl‐CoA for epigenetic modulation (Sun *et al*, 2019), storage of glycogen in brown adipose fat mediated by gut microbiota (Li *et al*, 2021), and glycogen accumulation in liver cancer aiding in liquid–liquid phase separation fueling tumorigenesis (Liu *et al*, 2021). New MALDI‐MSI techniques have demonstrated remarkable potential for early diagnosis, risk prediction, and treatment outcome for several cancers (Drake *et al*, 2017; Conroy *et al*, 2021; McDowell *et al*, 2021). Here, we utilize a new workflow for MALDI‐MSI *in situ* imaging of glycogen that provides spatial quantification of glycogen levels and architecture. Furthermore, this assay can be multiplexed with N‐linked glycans MALDI‐MSI. We applied the MALDI‐MSI method to quantify and visualize glycogen distribution in a number of organs in both mouse and human specimens as well as multiple cancers. This analysis revealed that ES, a pediatric bone cancer, not only exhibits high levels of glycogen stores, but ES glycogen also displays unique architecture that is more commonly observed in GSDs. Strikingly, targeting ES glycogen through genetic and pharmacological methods ablated *in vivo* tumor growth and identified this pathway as a novel therapeutic target.

Our MALDI‐MSI workflow allows simultaneous characterization of spatial and architectural glycogen heterogeneity *in situ*. Isoamylase treatment provides highly specific digestion of glucose chains from glycogen in FFPE tissue that results in quantification of released glucose polymers by time‐of‐flight mass spectrometry. Additionally, samples can be multiplexed using isoamylase treatment with PNGase F to simultaneously determine glycogen and N‐linked glycans on the same slide with the aid of ion mobility separation by collision cross section. This method will be a significant tool in understanding the microenvironmental metabolic interplay in both normal and diseased human clinical tissues. In addition to direct imaging of glycogen, this workflow also includes the in‐depth analysis of glycogen architecture such as chain length distribution and phosphate levels. These key parameters provide functional information on the nature of glycogen metabolism in the host tissue. It is worth noting that spatial glycogen could be affected by the fact that glycogenolysis has been shown to occur postmortem; therefore, euthanasia parameters should be taken into consideration when interpreting glycogen metabolism *in situ*. Rapid dissection and tissue fixation following euthanasia are critical for this analysis. Using the Waters Synapt G2 XS mass spectrometer, we can perform spatial analysis with a laser spot size of 50 μm that offers exceptional anatomical and regional glycogen distribution. The average human cell ranges from 10 to 100 μm (Gillooly *et al*, 2015; Ginzberg *et al*, 2015) and newer MALDI‐MSI mass spectrometers that offer 5 μm would allow near single‐cell analysis using our workflow.

We applied our MALDI‐MSI method to visualize glycogen distribution in a number of organs in both mouse and human specimens. Glycogen spatial distribution and relative abundance were defined in the liver, kidney, testis, lung, and bladder in wild‐type mice and liver, lung, brain, and the marrow and outer calcified layer of tibia from human tissues. While these organs all accumulate glycogen, regional glycogen distribution is heterogeneous between different tissue layers within each organ. For example, glycogen is preferentially accumulated in the urothelial lining rather than smooth muscle layer of the mouse bladder. Similarly, while both gray and white matter regions accumulate glycogen in the frontal cortex of the human brain, glycogen levels are substantially higher in the gray matter. Another interesting site of glycogen accumulation is the endothelium lining of blood vessels evident in the brain and liver of human specimens. These unique cellular layers that accumulate glycogen all have different functions. For example, the urothelium is a structural cell layer with tight junction that act as a barrier for re‐absorption of toxic material and pathogens by the bloodstream (Balsara & Li, 2017). These unique patterns of glycogen distribution suggest regional and cell‐specific roles of glycogen that remain to be elucidated.

In addition to normal tissue, we applied the glycogen imaging workflow to prostate cancer tissue with ductal corpora amylacea, LUSC, LUAD, and ES. We observed very low levels of intratumoral glycogen in prostate cancer and LUSC. Higher glycogen levels were only observed as part of ductal corpora amylacea of prostate and stromal, and blood vessels within the LUSC tumors. Conversely, LUAD and ES tissues exhibited extremely elevated levels of glycogen. Glycogen from these tissues exhibited hyperphosphorylation and hyperbranching, suggesting a common mechanism driving aberrant glycogen metabolism in these diseases. Perturbations in glycogen metabolism is a growing theme among a subset of cancers. We recently identified that aberrant nuclear glycogen metabolism in NSCLC results in epigenetic changes and increased cancer growth in mouse models (Sun *et al*, 2019). Additionally, liquid phase separated glycogen (presumably high in phosphate) is a critical driver of both tumorigenesis and proliferation in a mouse model of liver cancer (Curtis *et al*, 2019; Wolfe *et al*, 2021). These findings highlight an emerging appreciation for the role for glycogen in cancer; still, additional work is needed to rigorously identify classes of tumors that will respond to therapeutic agents targeting glycogen.

Chromosomal translocation is a hallmark of ES with around 85% of tumors harboring a t(11:22) (q24:q12) resulting in the EWS‐FLI1 oncoprotein. The contribution of EWS‐FLI1 and other ES chimeric proteins to tumorigenesis and metabolism has been extensively studied and reviewed (Lessnick & Ladanyi, 2012; Cidre‐Aranaz & Alonso, 2015; Grunewald *et al*, 2018; Sen *et al*, 2018; Riggi *et al*, 2021). ES is one of a few tumors that display profound PAS+ staining during histopathological analyses, demonstrated to be glycogen through an additional step of diastase treatment (Schajowicz, 1959; Telles *et al*, 1978). In fact, glycogen metabolic enzymes are drastically altered by EWS‐FLI1 (Tanner *et al*, 2017; Aynaud *et al*, 2020), including the glycogen phosphorylase muscle isoform (*PYGM*; Yin *et al*, 2018). The MALDI‐MSI method confirms extensive glycogen accumulation in ES tumors. We proceeded to demonstrate that excess glycogen is observed in ES resections from multiple tissue locations throughout the body. An interesting aspect of this analysis is identification of glycogen hyperphosphorylation as a hallmark of ES. ES glycogen closely resembles pathogenic PGBs observed in many GSDs. Strikingly, decreased protein expression of laforin is observed in ES tumors, and low laforin gene expression is associated with poorer overall survival of ES patients. In the case of the fatal childhood dementia Lafora disease, hyperphosphorylated glycogen aggregates are the etiological driver of rapid neurodegeneration and cognitive decline within the second decade of the patient's life (Gentry *et al*, 2018). Current work on Lafora disease is acutely focused on developing preclinical therapeutic candidates targeting these aberrant glycogen aggregates by enzymatic, antisense oligonucleotide, and pharmacological modalities (Austin *et al*, 2019; Brewer *et al*, 2019; Zhou *et al*, 2019; Tang *et al*, 2020). Multiple groups have demonstrated that downregulation of glycogen synthesis or enzymatic ablation of the aggregates rescues several Lafora disease phenotypes in mouse models (Brewer *et al*, 2019; Gumusgoz *et al*, 2021; Nitschke *et al*, 2021). Paralleling similar modalities as GSDs, we demonstrate both genetic and pharmacological targeting of glycogen accumulation or glycogen hyperphosphorylation blunted tumor growth *in vivo* in ES model cell lines. Our data support that aberrant glycogen is a clinical hallmark of ES, and with further research and development, glycogen could be a viable therapeutic target for precision therapy of ES.

In summary, our workflow provides a sensitive and precise method to interrogate glycogen structure and distribution *in situ*. We demonstrated the unique distribution of glycogen in multiple nondiseased and diseased tissues and the glycogen dependency of the pediatric cancer, Ewing sarcoma. Future experiments should include elucidation of the metabolic interplay between high and low glycogen cellular and tissue layers and how they contribute to overall physiology on an organismal level. Glycogen has been reported in multiple distinct cellular organelles or compartments, and continuing innovation of new technologies is needed to define the connection of glycogen and other cellular processes.

## Materials and Methods

### Chemicals and reagents

High‐performance liquid chromatography‐grade acetonitrile, ethanol, methanol, water, alpha‐cyano‐4‐hydroxycinnamic acid (CHCA), and trifluoroacetic acid (TFA) were purchased from Sigma‐Aldrich. Histological‐grade xylenes were purchased from Spectrum Chemical. Citraconic anhydride for antigen retrieval was obtained from Thermo Fisher Scientific. Recombinant PNGaseF Prime was obtained from N‐Zyme Scientifics (Doylestown, PA, USA). Isoamylase (Glycogen 6‐glucoanohydrolase) was purchased from Megazyme (Wicklow, Ireland). Guaiacol was purchased from Cayman Chemical (Ann Arbor, MI).

### Experimental model and subject details

A673 cells were purchased from ATCC and maintained in high‐glucose DMEM media supplemented with 10% FBS, 5 mM sodium pyruvate, and 5 mM L‐glutamine. TC32 cell line were generously gifted from Dr. Marko Leggas. Glycogen synthase knockout (GYS KO) cell lines were generated by Synthego Corporation. Growth rates of GYS KO cells were determined by plating 5,000 cells per well in four 96‐well plates for 3 days. Cell viability was determined by the neutral red cell viability assay as described (Repetto *et al*, 2008). For the generation of laforin‐OE cell lines, cells were infected with lentivirus carrying flag‐tagged laforin and selected for by puromycin (2 μg/ml). Flag‐tagged laforin lentiviral plasmid was purchased from Genecopoeia (EX‐U1135‐Lv102). Bone marrow‐derived mesenchymal stem cells were purchased from ATCC and maintained in mesenchymal stem cell basal medium supplemented with the FBS, L‐alanyl‐L‐Glutamine, EGF, FGF acidic and basic. Athymic Foxn1nu/(Nude) mice from Jackson Laboratory. Mice were housed in climate‐controlled environment with a 14/10 h light/dark cycle with water and solid diet provided *ad llibitum* throughout the study. The University of Kentucky Institutional Animal Care and Use Committee has approved all the animal procedures carried out under PHS #A3336‐01.

### Western blotting

Whole‐cell extracts were generated in 2% SDS in 50 mM Tris–HCl pH 7.5, and lysates were cleared by centrifugation. Protein concentrations were quantified by the Pierce BCA Protein Assay Kit (Thermo, Waltham, MA, USA). For Western blotting, 25 μg of protein extract per sample was denatured with heat and reducing agents, separated on a 4–12% acrylamide gel (Bio‐Rad), and transferred to PVDF membrane (Bio‐Rad). Antibodies for Western blotting recognized laforin (Abcam ab129110, 1:1,500) and rhodamine‐conjugated anti‐actin antibody (Bio‐Rad, 1:3,000). All antibodies have detailed species validation available online from vendors. The StarBright Blue 700 fluorescent secondary anti‐rabbit IgG antibody (Bio‐Rad, 1:3,000) was incubated for 1 h at room temperature. After washing, fluorescent images were acquired using a ChemiDoc MP (Bio‐Rad). Western blots were performed a minimum of three independent experiments and the results are representative of a single experiment.

### Immunohistochemistry

Mice were sacrificed by cervical dislocation followed by immediate tissue resection and fixation in neutral‐buffered 10% formalin (NBF) then paraffin embedded and stored prior to use. Fixed mouse tissue were sectioned at 4 μm immunohistochemistry was performed at the University of Kentucky Biospecimen Procurement and Translation Pathology Shared Resource Facility using the method previously described (Zhang *et al*, 2017). Antibodies used for immunohistochemistry recognized laforin, *EPM2A* (abcam), cleaved caspase 3 (abcam) and Ki67 (abcam). Digital images were acquired through the ZEISS Axio Scan.Z1 high‐resolution slide scanner. Image analysis was performed using the HALO software (Indica labs). Cleaved caspase 3 staining intensity was quantified by Multiplex IHC image analysis platform (v3.1) was used to quantify stain intensity and cell count. Ki‐67 mitotic indices were determined using the Multiplex IHC image analysis platform and visual confirmation of indices.

### Tissue procurement

De‐identified human patient tissues were obtained from the University of Kentucky Biospecimen Procurement and Translation Pathology Shared Resource Facility, the Sanders Brown Center on Aging and the Cooperative Human Tissue Network. All Ewing sarcoma samples were of primary tumor origin from different individuals. All tissues were preserved in neutral‐buffered formalin and then paraffin embedded for storage. All specimens conform to the principles set out in the WMA Declaration of Helsinki and the Department of Health and Human Services Belmont Report.

### Tissue preparation and enzyme digestion

Formalin‐fixed paraffin‐embedded (FFPE) blocks were sectioned at 4 μm, mounted on positively charged glass slides and processed similar as described (Powers *et al*, 2014; Stanback *et al*, 2021). In brief, slides were heated at 60°C for 1 h. After cooling, tissue sections were deparaffinized by washing twice in xylenes (3 min each). Tissue sections were then rehydrated by submerging the slide twice in 100% ethanol (1 min each), once in 95% ethanol (1 min), once in 70% ethanol (1 min), and twice in water (3 min each). Mesenchymal stem cells were at 30,000 per well and grown overnight. Cells were washed twice with 1× PBS, and fixed for 3 h in 10% neutral‐buffered formalin, and 70% ethanol for 2 h. The slides were transferred to a coplin jar containing the citraconic anhydride buffer for antigen retrieval and the jar was placed in a vegetable steamer for 25 min. Citraconic anhydride (Thermo) buffer was prepared by adding 25 ml citraconic anhydride in 50 ml water and adjusted to pH 3 with HCl. After allowing the buffer to cool, the buffer was exchanged with water five times by pouring out half of the buffer and replacing with water, prior to replacing completely with water on the last exchange. The slide was then desiccated prior to enzymatic digestion. Tris buffer pH 9–10 was also effective, but citraconic anhydride buffer was used for all experiments in this study.

### Glycogen and N‐glycan MALDI mass spectrometry imaging

An HTX spray station (HTX) was used to coat the slide with a 0.2 ml aqueous solution of either isoamylase (3 units/slide), PNGase F (20 mg total/ slide), or both. The spray nozzle was heated to 45°C and the spray velocity was 900 m/min. Following application of PNGase F, slides were incubated at 37°C for 2 h in a humidified chamber, then dried in a desiccator prior to matrix application [α‐cyano‐4‐hydroxycinnamic acid matrix (0.021 g CHCA in 3 ml 50% acetonitrile/50% water and 12 μl 25%TFA) applied with HTX sprayer]. For the detection and separation of glycogen and N‐glycans, a Waters Synapt G2 SX high‐definition mass spectrometer equipped with traveling wave ion mobility was used. The laser was operating at 1,000 Hz with an energy of 200 AU and spot size of 50 μm, mass range is set at 500–3,000 *m/z*. Images of glycogen and N‐glycans were generated using the waters HDI software.

Mouse liver sample sent to the Medical University of South Carolina (MUSC) was digested by isoamylase and chain length ions were detected using a trapped ion mobility time‐of‐flight mass spectrometer (timsTOF fleX; Bruker Daltonics, Bremen, Germany). The sample was analyzed in positive ion mode with a mass range from 700 to 3,500 *m/z*. The SmartBeam 3D 10 kHz laser was set to 45% power and a laser spot size of 20 μm. The images were collected with 300 shots per pixel and a 40 μm step size in between pixels. Following MS acquisition, the data were imported into SCiLS Lab 2022b Pro (Bruker Daltonics) and normalized to total ion current. Images of chain length glycogen were generated to view the expression pattern of each analyte of interest.

### Hydrolysis of glycogen to monomeric sugars

Mice were sacrificed by cervical dislocation, tumors were resected immediately postmortem, and washed once with PBS, twice with diH_2_O, blotted dry, and snap frozen in liquid nitrogen. The frozen tissues were pulverized to 5 μm particles in liquid nitrogen using a Freezer/Mill Cryogenic Grinder (SPEX SamplePrep). Pulverized tissue (20 mg) was extracted in 50% methanol/chloroform (V/V 1:1) and separated into polar (aqueous layer), lipid (chloroform layer), and protein/glycogen (interfacial layer) fractions. Glycogen was separated from the protein pellet using 1.5 ml of ice cold 10% trichloroacetic acid. Isolated glycogen was dried by vacuum centrifuge at 10^−3^ mBar. Hydrolysis of glycogen was performed by first resuspending the pellet in diH_2_O followed by the addition equal parts 4 N HCl. Samples incubated at 95°C for 2 h on a vortex mixer with a heated lid. The reaction was quenched with 100% methanol with 40 μM L‐norvaline (as an internal control). The sample was then incubated on ice for at least 30 min. The supernatant was collected by centrifugation at 15,000 rpm at 4°C for 10 min and subsequently dried by vacuum centrifuge at 10^−3^ mBar.

### Gas chromatography mass spectrometry (GCMS) analysis

Dried samples were derivatized by the addition of 20 mg/ml methoxyamine hydrochloride in pyridine and incubation for 1.5 h at 30°C. Sequential addition of N‐methyl‐trimethylsilyl‐trifluoroacetamide (MSTFA) followed with an incubation time of 30 min at 37°C with thorough mixing between addition of solvents. The mixture was then transferred to a v‐shaped amber glass chromatography vial. An Agilent 7800B gas‐chromatography (GC) coupled to a 7010A triple quadrupole mass spectrometry detector equipped with a high‐efficiency source was used for this study. GCMS protocols were similar to those described previously (Sun *et al*, 2019; Young *et al*, 2020), except a modified temperature gradient was used for GC: Initial temperature was 130°C, held for 4 min, rising at 6°C/min to 243°C, rising at 60°C/min to 280°C, held for 2 min. The electron ionization (EI) energy was set to 70 eV. Scan (*m/z*: 50–800), and full scan mode were used for target metabolite analysis. Metabolite EI fragmentation pattern and retention time were determined by ultrapure standard purchased from sigma. Ions (*m/z*) and retention time (min) used for glycogen quantitation was glucose (160 or 319 *m/z*; 17.4 min), Relative abundance was corrected for recovery using the L‐norvaline standard and adjusted to protein input represented by pooling amino acids detected by GCMS (Andres *et al*, 2020).

### Xenograft

For *in vivo* tumor growth, A673, TC32, and TC71 cells were counted and resuspended at 1 × 10^6^ cells per 100 μl of PBS. Female 5–7‐week‐old Foxn1nu/(Nude) mice (JAX Laboratories) were injected subcutaneously with 1 × 10^6^ cells in each of the flanks. Tumor growth was measured every 3 days by caliper in a nonblinded fashion. For glycogen synthase inhibitor injections, intraperitoneal injections daily (Monday–Friday) occurred. Tumors were allowed to reach 2,000 mm^3^ and then mice were sacrificed. All of the mouse experiments were approved by the Animal Care and Use Committee and the University of Kentucky Institutional Animal Care and Use Committee (IACUC). The committees are both accredited by the Association for Assessment and Accreditation of laboratory Animal Care and were performed in accordance with institutional and national regulations and guidelines.

### Gene expression analysis

Retrospective analysis of laforin (EPM2A) expression was performed utilizing GEO Accession viewer and BIOGPS. Gene expression data for EPM2A GSE17679, GEOD‐17674, and GEOD‐37371 were downloaded and expressed as relative expression.

### Statistics

Statistical analyses were carried out using GraphPad Prism. Statistical analysis can be found in each figure caption. All numerical data are presented as mean ± SEM except for xenograft tumor growth, which is presented as mean ± SD. Grouped analysis was performed using one‐way ANOVA with Tukey's multiple comparison or two‐way ANOVA with Tukey's multiple comparison. Column analysis was performed using one‐way ANOVA or *t*‐test. A *P*‐value < 0.05 was considered statistically significant.

## Author contributions

**Lyndsay EA Young:** Data curation; investigation; methodology; writing – original draft; writing – review and editing. **Lindsey R Conroy:** Data curation; investigation; writing – review and editing. **Harrison A Clarke:** Data curation; investigation. **Tara R Hawkinson:** Data curation; investigation; methodology. **Kayli E Bolton:** Investigation. **William C Sanders:** Investigation. **Josephine E Chang:** Investigation. **Madison B Webb:** Investigation. **Warren J Alilain:** Investigation. **Craig W Vander Kooi:** Formal analysis; supervision; writing – review and editing. **Richard R Drake:** Resources; investigation; methodology. **Douglas A Andres:** Resources; investigation. **Tom C Badgett:** Resources. **Lars M Wagner:** Resources. **Derek B Allison:** Resources; formal analysis; investigation. **Ramon C Sun:** Conceptualization; resources; supervision; writing – original draft; project administration. **Matthew S Gentry:** Conceptualization; resources; writing – original draft; project administration.

## Disclosure and competing interests statement

RCS has research support and received consultancy fees from Maze Therapeutics. RCS is a co‐founder of Attrogen LLC. RCS is a member of the Medical Advisory Board for Little Warrior Foundation. DBA received book royalty from Wolters Kluwer. MSG has research support and research compounds from Maze Therapeutics, Valerion Therapeutics, Ionis Pharmaceuticals. MSG also received consultancy fee from Maze Therapeutics, PTC Therapeutics, and the Glut1‐Deficiency Syndrome Foundation. MSG is a co‐founder of Attrogen LLC.

**For more information**

- https://littlewarrior.org/
- https://www.ncbi.nlm.nih.gov/geo/query/acc.cgi?acc=gse17679
- https://www.ncbi.nlm.nih.gov/geo/query/acc.cgi?acc=GSE17674
- https://www.ncbi.nlm.nih.gov/geo/query/acc.cgi?acc=GSE37371

## Supporting information




Appendix

## Data Availability

All data are available in the main text or the supplementary materials. This study includes no data deposited in external repositories.
