# Supplementary material for: In situ mass spectrometry imaging reveals heterogeneous glycogen stores in human normal and cancerous tissues
Source: EMBO Mol Med. 2022 Sep 5;14(11):e16029. doi: 10.15252/emmm.202216029 (PMC9641418; doi:10.15252/emmm.202216029)
Supplement: Supplementary file 1 — Appendix [file EMMM-14-e16029-s001.pdf]

Supplementary Materials for

In situ mass spectrometry imaging reveals heterogeneous glycogen stores in human normal and cancerous tissues

Lyndsay E.A. Young[1, #], Lindsey R. Conroy[2,3, #], Harrison A. Clarke[2], Tara R. Hawkinson[2], Kayli E. Bolton[1], William C, Sanders[1], Josephine E. Chang[2], Madison B. Webb[1], Warren J. Alilain[2,6], Craig W. Vander Kooi[1,3], Richard R. Drake[4], Douglas A. Andres[1], Tom C. Badgett[7], Lars M. Wagner[8], Derek B. Allison[5], Ramon C. Sun* [2,3,6] and Matthew S. Gentry* [1,3]

*Correspondence: ramon.sun@uky.edu or matthew.gentry@uky.edu

This PDF file includes:

Appendix Fig. S1 to S6 and legends

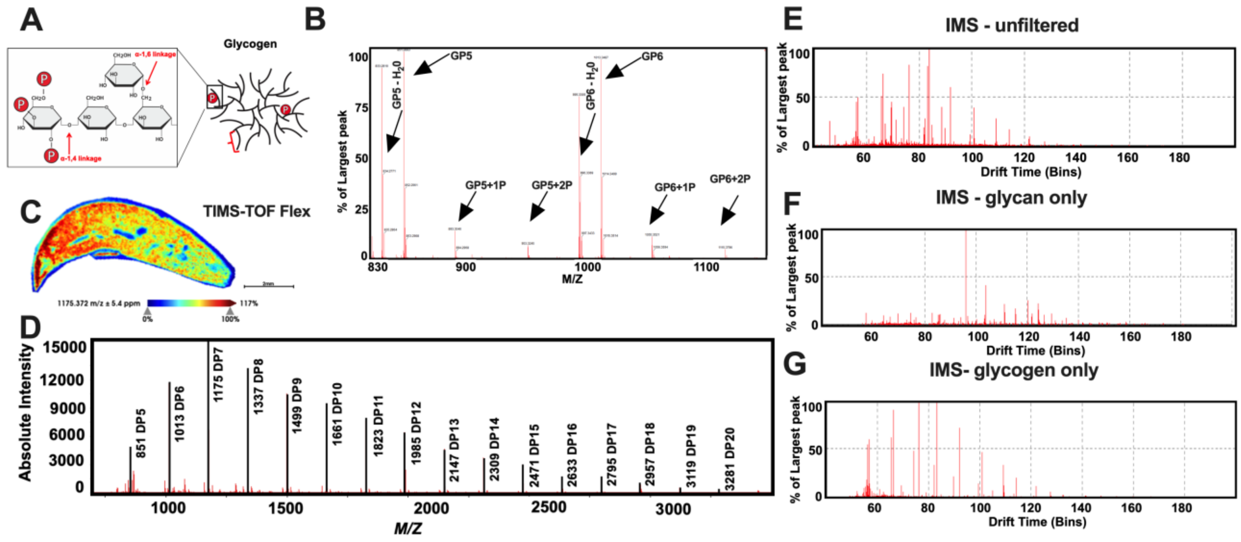

Fig. S1. MALDI-MSI of glycogen and N-linked glycans by traveling wave imaging mass spectrometry.

A. Schematic of glycogen structure demonstrating chain length alpha-1,4 linkages, alpha-1,6 bonds at branching points and covalently bonded phosphate to glucose hydroxyls.
B. Representative ion spectra of linear chain length/glucose polymer (GP) 5 and 6 and their respective phosphorylated forms detected by MALDI-TOF.
C. Validation of MALDI-MSI spatial distribution of glycogen (represented by CL7) in mouse liver section at the Medical University of South Carolina (MUSC) on a Bruker TIMS-TOF Flex. The image displays a heatmap gradient of intensity with blue (least abundant) to red (most abundant).
D. Relative abundance of *m/z* extracted from the glucose polymers of the mouse liver representing glycogen chain lengths ranging from *m/z*=750-4000.
E. Total ion spectra before application of ion mobility separation showing mixture of glucose polymer chains and N-linked glycans after isoamylase and PNGase F digestion.
F. 1D plot of draft time showing N-linked glycans migration through Ion mobility separation (IMS) from ion spectra of **D**.
G. 1D plot of draft time showing N-linked glycans migration through Ion mobility separation (IMS) from ion spectra of **D**.

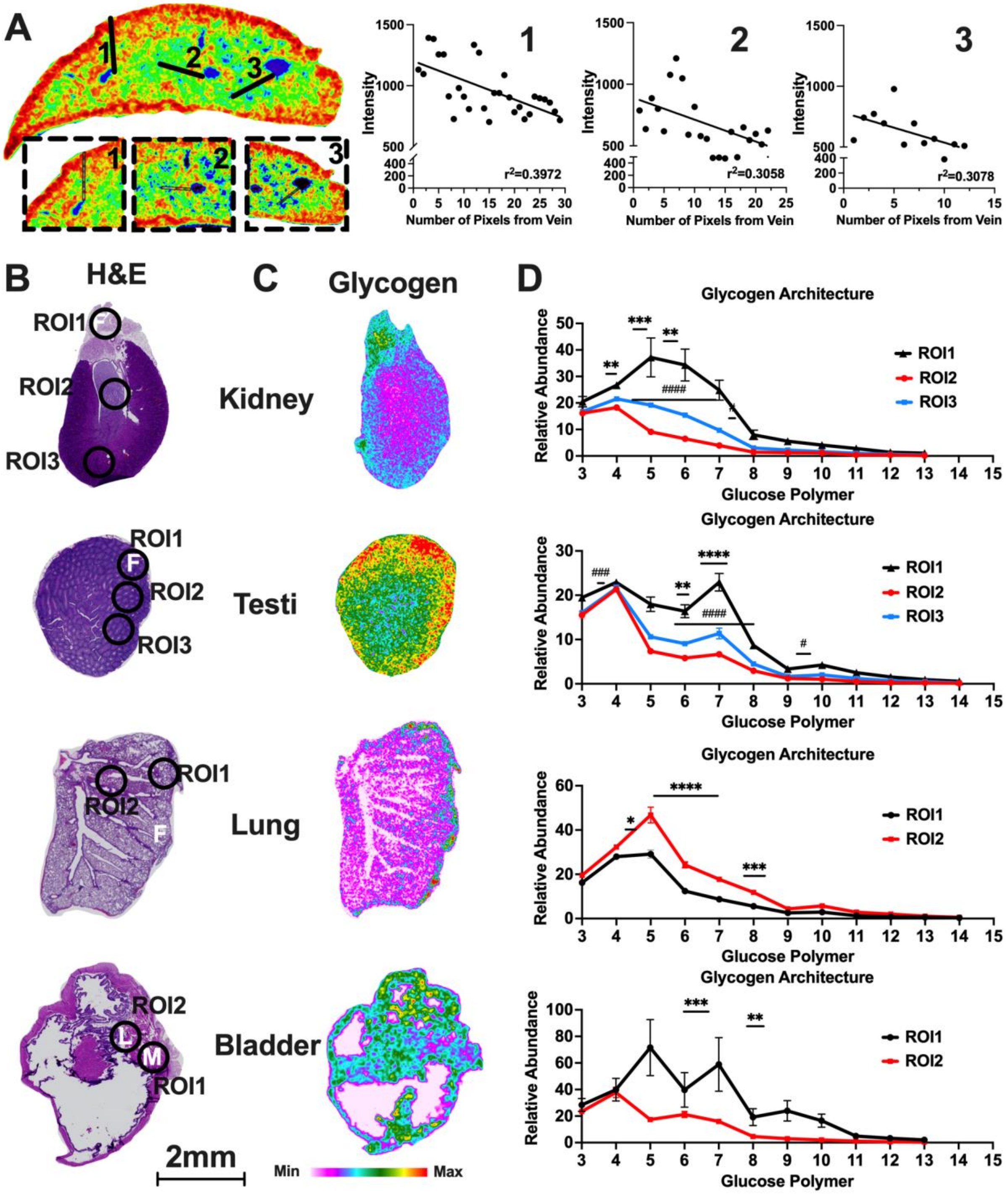

Fig. S2. Spatial analysis of glycogen structure across mouse kidney, testi, lung, and bladder tissues.

A. (*Left*) Spatial distribution and relative abundance of glycogen (represented by CL7) from the mouse liver section showing three quantified pixel gradients from the central vein to Glisson's capsule. (*Right*) Linear regression analysis of pixel distance compared to total glycogen relative

abundance for each gradient. Values are presented as relative abundance for pixels within each region. Image is of the same tissue shown in **Fig. 1G** and **Fig. 2B**.

B. Hematoxylin and eosin (H&E) stained cross section of normal mouse kidney, testis, lung, and bladder tissues from a wild type mouse. Annotated regions are the F-fibrous layer, L-lining layer, and M-muscle and ROIs were based on anatomy. Scale bar is represented below the images for **B** and **D**.

C. Spatial distribution and relative abundance of glycogen (represented by CL7) from an immediate adjacent resected tissue section shown in **A**. The image displays a heatmap gradient of intensity with white (least abundant) to red (most abundant).

D. Relative abundance of m/z extracted from the glucose polymer regions from **A** representing glycogen chain lengths ranging from *m/z*=500-3500. Values are presented as mean +/- standard error (n=3 technical replicates per region).

$^{*/\#}0.01 < P < 0.05$; $^{**/\#\#}0.001 < P < 0.01$; $***^{/\#\#\#}$ $P < 0.001$; $****^{/\#\#\#\#}P<0.0001$, analyzed by one-way ANOVA (three ROIs) or two-tailed t-test (two ROIs) for each glycogen chain length.

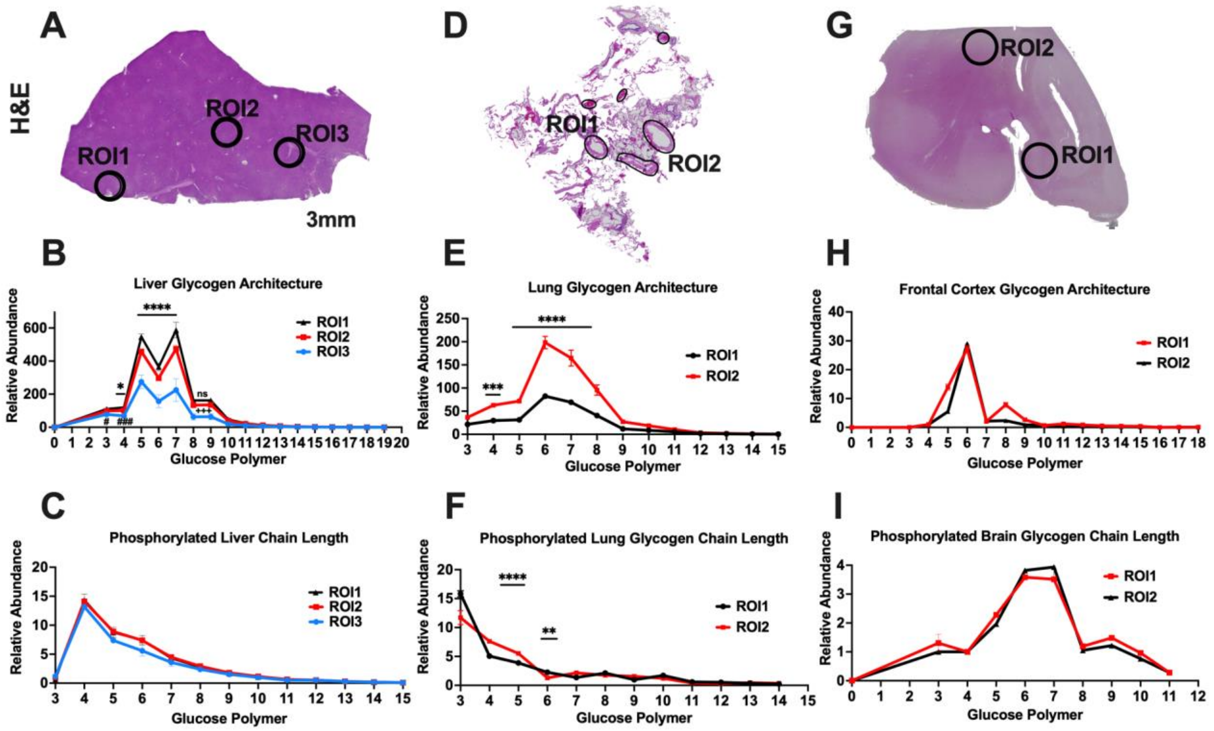

Fig. S3. Spatial analysis of normal human liver, lung, and brain glycogen structure.

A. Hematoxylin and eosin (H&E)-stained cross-section of a normal human liver. Three separate regions were extracted for regional structural analysis. Glisson's capsule (ROI1), endothelium lining of the central vein (ROI2), and hepatocyte nodule (ROI3). Image is also used in **Fig. 3A**.

B. Glycogen structure defined by distribution of released glucose polymers as representation of glycogen chain length (CL) distribution from each ROI in **A.** $^{*/\#}0.01 < P < 0.05$; $***^{/\#\#\#}$ $P < 0.001$; $****P<0.0001$, analyzed by one-way ANOVA with Tukey's multiple comparison for each glycogen chain length.

C. Phosphorylated glycogen structure defined by distribution of phosphorylated glucose polymers as representation of chain length distribution of the ROIs in **A**.
D. H&E-stained cross-section of a normal human lung. Annotated regions are blood vessels found in the airway, and lung alveoli (ROI1) and endothelium lining of blood vessels (ROI2). Image is also used in **Fig. 3C**.
E. Glycogen structure defined by distribution of released glucose polymers as representation of chain length distribution for each ROI in **D**. ***P < 0.001; ****P<0.0001, analyzed by two-tailed t-test for each glycogen chain length.
F. Phosphorylated glycogen structure defined by distribution of phosphorylated glucose polymers as representation of chain length distribution of the ROIs in **D**. **P < 0.01; ****P<0.0001, analyzed by two-tailed t-test for each glycogen chain length.
G. H&E-stained cross-section of aged human brain tissue. Annotated regions are grey matter (ROI1) and white matter (ROI2). Image is also used in **Fig. 3E**.
H. Glycogen structure defined by distribution of released glucose polymers as representation of glycogen chain length (CL) distribution from each ROI in **G.**
I. Phosphorylated glycogen structure defined by distribution of phosphorylated glucose polymers as representation of chain length distribution of the ROIs in **G**.
Values are presented as mean +/- standard error (n=3 technical replicates per region) for **B-I**.

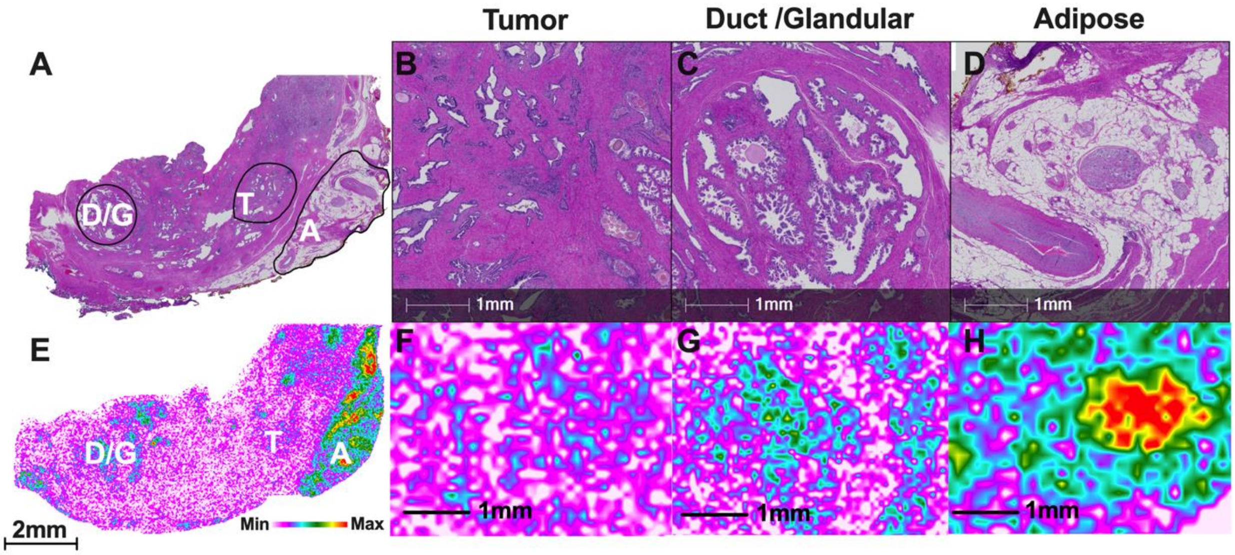

Fig. S4. Spatial analysis of glycogen structure of grade group 1 prostate tumor tissue.
A. Hematoxylin and eosin (H&E) stained cross section of grade 1 prostate cancer. Annotated regions are annotated as T-tumor, D/G-duct/glandular, and A-adipose tissue. Scale bar is represented below the images. Image is also used in **Fig. 4A**.
B-D. Magnified H&E cross section of tumor (**B**), duct/glandular (**C**), and adipose (**D**) tissues.
E. Spatial distribution and relative abundance of glycogen (represented by CL7) from an immediate adjacent resected tissue section shown in **A**. The image displays a heatmap gradient of intensity with white (least abundant) to red (most abundant). Image is also used in **Fig. 4B**.

F-H. Magnified MALDI-MSI image of glycogen in tumor (**F**), duct/glandular (**G**), and adipose (**H**) tissues. The image displays a heatmap gradient of intensity with white (least abundant) to red (most abundant).

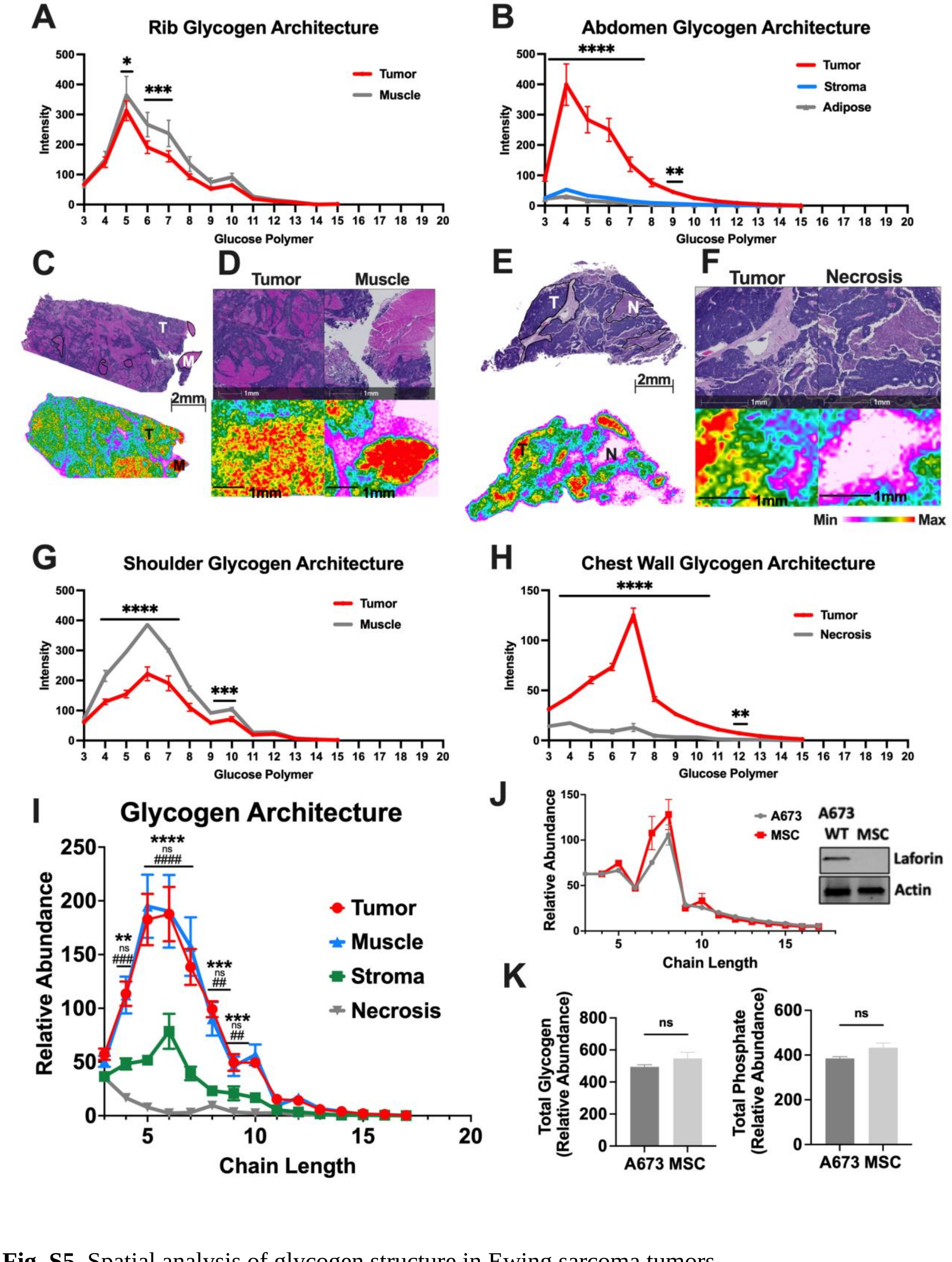

Fig. S5. Spatial analysis of glycogen structure in Ewing sarcoma tumors.

A. Glycogen structure defined by distribution of released glucose polymers as representation of glycogen chain length distribution of tumor and muscle tissue of the Ewing sarcoma (ES) of the rib tumor in **Fig. 5C**. Values are presented as mean +/- standard error (n=3 technical replicates per region). $^{*}0.01 < P < 0.05$; ***P < 0.001, analyzed by one-way ANOVA with Tukey's multiple comparison for each glycogen chain length.
B. Glycogen structure defined by distribution of released glucose polymers as representation of glycogen chain length distribution of tumor, stroma, and adipose tissue of the ES of the abdomen tumor in **Fig. 5D**. Values are presented as mean +/- standard error (n=3 technical replicates per region). ***P < 0.001; ****P < 0.0001, analyzed by one-way ANOVA with Tukey's multiple comparison for each glycogen chain length.
C. (*Top*) Hematoxylin and eosin (H&E) stained cross section of the ES of the shoulder tumor in **Fig. 5A**. Annotated regions are annotated as T-tumor and M-muscle. (*Bottom*) Spatial distribution and relative abundance of glycogen (represented by CL7) from an immediate adjacent resected tissue section. Images are also used in **Fig. 5A** and **S6D**.
D. Magnified H&E-stained cross-section of (*top, left)* tumor and (*top, right*) muscle tissues with magnified image of CL7 (*m/z*=1175) of (*bottom, left)* tumor and (*bottom, right*) muscle tissues.
E. (*Top*) H&E-stained cross-section of the ES of the chest wall tumor in **Fig. 5B**. Annotated regions are annotated as T-tumor and N-necrosis. (*Bottom*) Spatial distribution and relative abundance of glycogen (represented by CL7) from an immediate adjacent resected tissue section. The image displays a heatmap gradient of intensity with white (least abundant) to red (most abundant). Scale bar is represented below the images. Images are also used in **Fig. 5B** and **S6E**.
F. Magnified H&E cross section of (*top, left)* tumor and (*top, right*) necrosis tissues with magnified image of CL7 *(m/z*=1175) of (*bottom, left)* tumor and (*bottom, right*) necrosis tissues
G. Glycogen structure defined by distribution of released glucose polymers as representation of glycogen chain length distribution of tumor and muscle tissue of the Ewing sarcoma (ES) of the shoulder tumor in **C** and **D**. Values are presented as mean +/- standard error (n=3 technical replicates per region). ***P < 0.001; ****P < 0.0001, analyzed by two-tailed t-test for each glycogen chain length.
H. Glycogen structure defined by distribution of released glucose polymers as representation of glycogen chain length distribution of tumor and necrosis tissue of the Ewing sarcoma (ES) of the chest wall tumor in **F** and **G**. Values are presented as mean +/- standard error (n=3 technical replicates per region). **P < 0.01; ****P < 0.0001, analyzed by two-tailed t-test for each glycogen chain length.
I. Glycogen structure between ES tumors and multiple non-tumours regions in a pooled analysis using samples from **Fig. 5A-E**. Values are presented as mean +/- standard error (n=3 technical replicates per region). **P < 0.01; ***P < 0.001; ****P < 0.0001, analyzed by two-tailed t-test for each glycogen chain length. *: Tumor versus necrosis; #: Tumor versus stroma.
J. (*Left*) Glycogen structure defined by distribution of released glucose polymers as representation of glycogen chain length distribution in WT A673 Ewing sarcoma cells versus mesenchymal stem cells (MSC). Values are presented as mean +/- standard error (n=3 technical replicates per region). **P < 0.01; ****P < 0.0001, analyzed by two-tailed t-test for each glycogen chain length. (*Right*) Western blot of laforin expression in A673 and MSC.
K. MALDI-MSI quantitation of (*left*) total glycogen and (*right*) total phosphate abundance in A673 and MSC, analyzed by two-tailed t-test. Values are presented as mean +/- standard error (n=3 biological replicates per group).

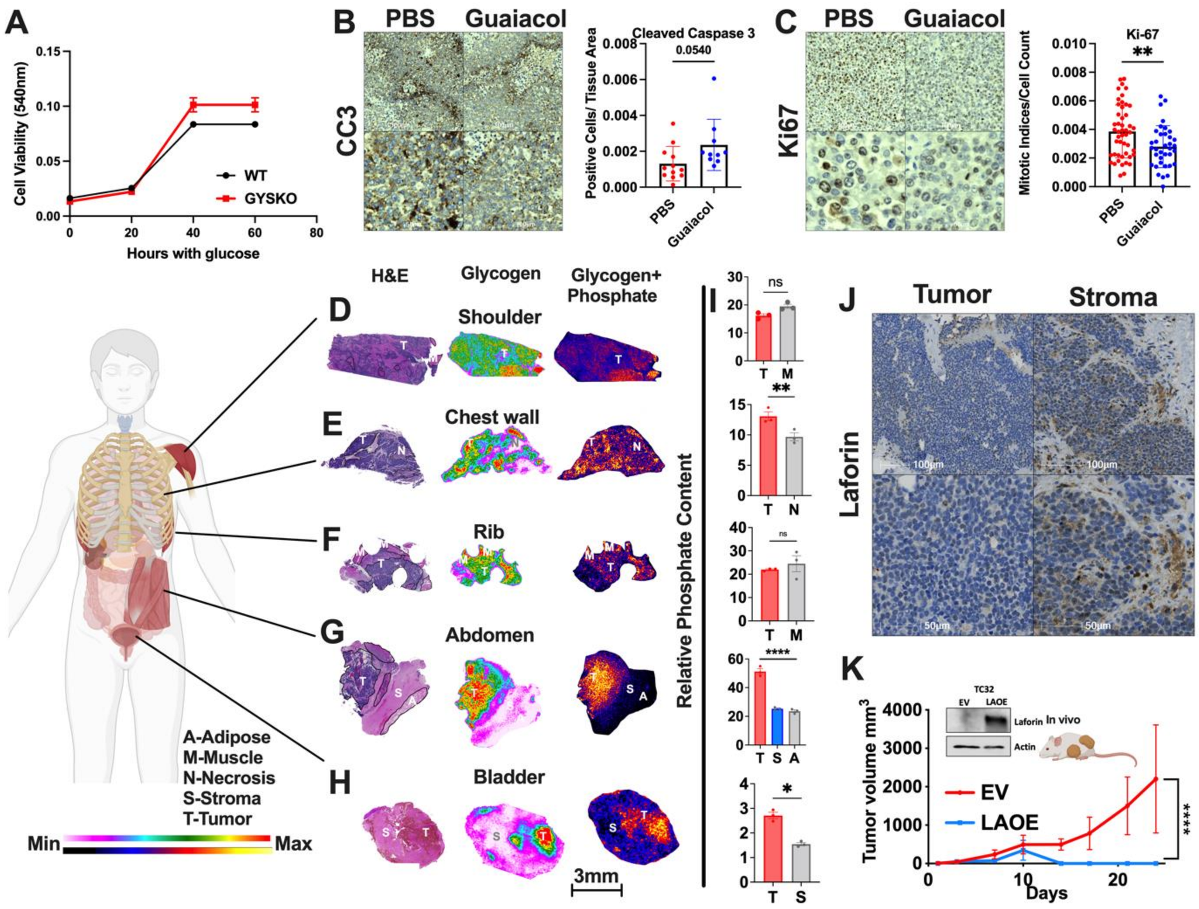

Fig. S6. Microenvironmental glycogen phosphate and laforin analysis of five Ewing sarcoma tumor tissues.

A. *In vitro* cell viability of A673 expressing either empty vector (EV) or glycogen synthase knockout (GYS1-KO) grown as monolayer. Values are presented as mean +/- standard error (n=3 biological replicates per group).

B. (*Left*) Magnified immunohistochemical staining of Cleaved Caspase 3 (CC3) in PBS and guaiacol-treated A673 tumors. Tissues were scanned digitally using the Axio Scan.Z1 side scanner. (*Right*) Quantification of immunohistochemical CC3 staining, analyzed by two-tailed t-test. Values are presented as mean +/- standard error (n=8 biological replicates per group).

C. (*Left*) Magnified Ki67 staining in PBS and guaiacol-treated A673 tumors. Tissues were scanned digitally using the Axio Scan.Z1 side scanner. (*Right*) Quantification of mitotic indices/cell. Values are presented as mean +/- standard error (n=8 biological replicates per group). $^{**}0.001 < P < 0.0,1$ analyzed by two-tailed t-test.

D. (*Left)* Hematoxylin and eosin (H&E)-stained cross section of Ewing sarcoma (ES) of the shoulder tumor. Annotated regions are annotated as T-tumor and M-muscle. (*Middle*) Spatial distribution and relative abundance of glycogen (represented by CL7) from an immediate adjacent resected tissue section. The image displays a heatmap gradient of intensity from white (least abundant) to red (most abundant). (*Right)* Spatial distribution and relative abundance of phosphorylated CL6. The image displays a heatmap gradient of intensity with black (least abundant) to yellow (most abundant). Scale bar and gradients are represented below the images for **A**-**E**. Images are also used in **Fig. 5A** and **S5C**.
E. (*Left)* H&E-stained cross section of ES of the chest wall tumor. Annotated regions are annotated as T-tumor and N-necrosis. (*Middle*) Spatial distribution and relative abundance of glycogen (represented by C:7) from an immediate adjacent resected tissue section. (*Right)* Spatial distribution and relative abundance of phosphorylated CL6. Images are also used in **Fig. 5B** and **S5E**.
F. (*Left*) H&E-stained cross section of ES of the rib tumor. Annotated regions are annotated as T-tumor and M-muscle. (*Middle*) Spatial distribution and relative abundance of glycogen (represented by CL7) from an immediate adjacent resected tissue section. (*Right)* Spatial distribution and relative abundance of phosphorylated CL6. Images are also used in **Fig. 5C.**
G. (*Left*) H&E-stained cross section of ES of the abdomen tumor. Annotated regions are annotated as T-tumor, S-stroma, and A-adipose tissue. (*Middle*) Spatial distribution and relative abundance of glycogen (represented by CL7) from an immediate adjacent resected tissue section. (*Right)* Spatial distribution and relative abundance of phosphorylated CL6. Images are also used in **Fig. 4A**, **4B**, and **5D**.
H. (*Left*) H&E-stained cross section of ES of the bladder tumor. Annotated regions are annotated as T-tumor, and S-stroma. (*Middle*) Spatial distribution and relative abundance of glycogen (represented by CL7) from an immediate adjacent resected tissue section. (*Right)* Spatial distribution and relative abundance of phosphorylated CL6. Images are also used in **Fig. 5E**.
I. MALDI-MSI quantitation of relative phosphate abundance of CL5, CL6, and CL7 for the annotated regions found in **D**-**H**, Values are presented as mean +/- standard error (n=3 technical replicates per region). $^{*}0.01 < P < 0.05$; $^{**}P < 0.01$; $^{****}P < 0.0001$ analyzed by two-tailed t-test for **D**-**H** or one-way ordinary ANOVA for **G**.
J. Magnified immunohistochemical staining of Laforin in ES of the bladder tumor (*left)* and stromal (*right)* tissues.
K. (*Top*): western blot showing confirmation of LAOE in the TC32 xenograft tumors. (*Bottom*): tumor growth of xenografts TC32 empty vector (EV) and laforin overexpression (LAOE). Values are presented as mean +/- standard error (n=8 biological replicates per group). ****P<0.0001, analyzed by two-tailed t-test.
