# Supplementary material for: In situ mass spectrometry imaging reveals heterogeneous glycogen stores in human normal and cancerous tissues
Source: EMBO Mol Med. 2022 Sep 5;14(11):e16029. doi: 10.15252/emmm.202216029 (PMC9641418; doi:10.15252/emmm.202216029)
Supplement: Supplementary file 2 [file EMMM-14-e16029-s002.pdf]

EMBO Molecular Medicine

In situ mass spectrometry imaging reveals heterogeneous glycogen stores in human normal and cancerous tissues

Lyndsay Young, Lindsey Conroy, Harrison Clarke, Tara Hawkinson, Kayli Bolton, William Sanders, Josephine Chang, Madison Webb, Warren Alillain, Craig Vander Kooi, Richard Drake, Douglas Andres, Tom Badgett, Lars Wagner, Derek Allison, Ramon Sun, and Matthew Gentry
DOI: 10.15252/emmm.202216029

Corresponding author: Ramon Sun (ramon.sun@uky.edu)

Review Timeline:

Submission Date:	22nd Mar 22
Editorial Decision:	14th Apr 22
Revision Received:	19th Jul 22
Editorial Decision:	21st Jul 22
Revision Received:	25th Jul 22
Accepted:	3rd Aug 22

Editor: Zeljko Durdevic

Transaction Report:

(Note: With the exception of the correction of typographical or spelling errors that could be a source of ambiguity, letters and reports are not edited. Depending on transfer agreements, referee reports obtained elsewhere may or may not be included in this compilation. Referee reports are anonymous unless the Referee chooses to sign their reports.)

14th Apr 2022

Dear Dr. Sun,

Thank you for the submission of your manuscript to EMBO Molecular Medicine. We have now received feedback from the three reviewers who agreed to evaluate your manuscript. As you will see from the reports below, the referees acknowledge the interest of the study but also raise important concerns that should be addressed in a major revision.

Further consideration of a revision that addresses reviewers' concerns in full will entail a second round of review. EMBO Molecular Medicine encourages a single round of revision only and therefore, acceptance or rejection of the manuscript will depend on the completeness of your responses included in the next, final version of the manuscript. For this reason, and to save you from any frustrations in the end, I would strongly advise against returning an incomplete revision.

We would welcome the submission of a revised version within three months for further consideration. Please let us know if you require longer to complete the revision.

Please use this link to login to the manuscript system and submit your revision: https://embomolmed.msubmit.net/cgi-bin/main.plex

I look forward to receiving your revised manuscript.

Yours sincerely,

Zeljko Durdevic

Zeljko Durdevic
Editor
EMBO Molecular Medicine

***** Reviewer's comments *****

Referee #1 (Comments on Novelty/Model System for Author):

The manuscript concerns the analysis of glycogen using imaging mass spectrometry. The authors report higher levels of glycogen in a number of tumors, of which the highest levels were found in Ewing's Sarcoma. It is then reported that interfering with glycogen synthase, the enzyme that builds glycogen, via CRISPR-Cas9 knockouts or using the inhibitor guaiacol, reduces tumor growth in Ewing Sarcoma. This Ewing Sarcoma part is by far the strongest part of the work, and clearly the approach could be applied to many other diseases to investigate the potential role of glycogen in their development. My main concern is the very observational nature of the data, and the fact that the numbers of subjects are not provided and the statistical analysis is far from clear, or far from satisfactory. For example. Figure 2C, the statistical test (one way ANOVA) were applied using pixel data from one imaging mass spectrometry dataset. It is not appropriate to use such tests on pixel data because the data is not independent...

Referee #1 (Remarks for Author):

The manuscript concerns the analysis of glycogen using imaging mass spectrometry. The authors report higher levels of glycogen in a number of tumors, of which the highest levels were found in Ewing's Sarcoma. It is then reported that interfering with glycogen synthase, the enzyme that builds glycogen, via CRISPR-Cas9 knockouts or using the inhibitor guaiacol, reduces tumor growth in Ewing Sarcoma. This Ewing Sarcoma part is by far the strongest part of the work, and clearly the approach could be applied to many other diseases to investigate the potential role of glycogen in their development. My main concern is the very observational nature of the data, and the fact that the numbers of subjects are not provided and the statistical analysis is far from clear, or far from satisfactory. For example, in Figure 2C, the statistical test (one way ANOVA) were applied using pixel data from one imaging mass spectrometry dataset. It is not appropriate to use such tests on pixel data because the data is not independent...

The manuscript is also currently too observational. Instead of many paired H&E and glycogen images, but in which any histological annotations have not been mapped onto the mass spectrometry images, it would be much more objective to extract the average signatures from specific histopathological regions, for multiple patient samples, and report the average levels in these regions, with appropriate statistical tests. i.e. please provide quantifiable data.

The section on simultaneous N-linked glycan and glycogen should not be included here, as it serves little purpose (never referred to later) and the authors did not demonstrate if/how it affects sensitivity. Using two enzymes will release both molecular types, but the added complexity of the molecular information is expected to lead to competition for charge (during ionization) and capacity if using ion trapping technologies (e.g. FTICR). Here the novelty is on glycogen imaging. This reviewer advises the authors to focus better, and develop a separate analytical investigation of this dual enzyme approach in which sensitivity is properly established.
Please improve figures, particularly Figure 1 (many screen grabs of insufficient resolution, and red and blue channels of 1G are not legible). Currently many panels in Figure 1 are barely comprehensible.

There is some evidence of publication bias in this manuscript. Referring to figure 1B on page 4 the authors describe "a gradient of glycogen stores radiating away from the central vein". This gradient is far from clear, and should be explicitly demonstrated by extracting the gradient data from the imaging mass spectrometry datasets, for multiple mouse liver samples (from different animals). Similarly in Figure 6A the the authors describe on page 6 the "normal bone tissue, glycogen predominantly accumulated in the bone marrow with less extent in the outer calcified bone". However the methodology is based on enzymatic activity of the isomyalase and the authors do not appear to have ever considered if the enzyme has equal access to all tissue types - in this instance enzyme is much less likely to access to glycogen encolsed in the bone... some critical analysis is lacking.

This reviewer is postive about the work, but the results need to be presented in a more quantitative manner, and the statistics more clearly described, especially the numbers of biological samples from which the conclusions were drawn.
Minor.
Figure 5C - the label M in the figure should be N (for Necrosis)
Figure 6B. Please define what high, outer, and low refer too as it is never defined.
Figure legends. Please provide legends that explain symbols used within the figures, so as to spare the reader from trawling through lengthy captions.
page 6. Please define what is meant by GSD,,,

Referee #2 (Comments on Novelty/Model System for Author):

I am not an expert in MALDI-MSI and therefore cannot really judge the technical quality of the corresponding experiments in detail. However, the demonstration of the versatility of the introduced technological pipeline to assess glycogen content, spatial distribution and architecture on FFPE slides of various tissues is convincing and the results are novel laying the basis for future functional studies. They also identify a new avenue to target Ewing sarcoma and may therefore impact patient treatment with an innovative approach. However, potential toxicity issues arising from treatment with glycogen biosynthesis targeting compounds are not addressed.

Referee #2 (Remarks for Author):

Glycogen is a key intracellular glucose metabolite involved in a still expanding number of physiological processes. Our knowledge on tissue and cell-type specific glycogen stores has so far largely been limited to its detectability by periodic acid-Schiff (PAS) reaction. In this manuscript, Young et al. utilize a novel matrix-assisted laser desorption/ionization mass spectrometry-based imaging pipeline for the analysis of FFPE sections to describe architectural and spatial heterogeneity in glycogen expression of normal mouse and human tissues, and to characterize quantitative and qualitative glycogen patterns in some cancers with a focus on Ewing sarcoma, a pediatric bone tumor characterized by high PAS positivity. For the first time, they report the spatial distribution of glycogen chains of distinct lengths, architecture and phosphate content with high resolution in tissues and cell types of major organs, prostate cancer, non-small cell lung cancer and Ewing sarcoma. Importantly, by genetic (CRISPR/Cas9) and pharmacologic knockout/knockdown of glycogen synthetase GYS1 or ectopically forcing expression of glycogen phosphatase laforin, they provide evidence that targeting the glycogen biosynthetic pathway significantly reduces Ewing sarcoma growth in vivo. These findings may open new therapeutic avenues for the treatment of this devastating disease.

Specific comments:
Figure S1C: To illustrate robustness of the method across different MALDI-MSI platforms, Figure S1C presents MALDI-MSI spatial distribution of glycogen in a rat brain using a Bruker FTICR mass spectrometer, and text says that "a similar chain length distribution was observed". Similar to what? To the results obtained with the Waters Synapt G2 XS ion mobility-enabled mass spectrometer? These are not shown for mouse brain. Or to mouse liver, which is discussed in the preceding lines?

Figure S3C, S3F, S3I: Text says that phosphorylated chain length in non-diseased tissues displayed similar polymeric distribution compared to non-phosphorylated glycogen species. However, it appears that for liver and lung, abundance of phosphorylated polymers was highest at a chain length of around 4, and for brain frontal cortex it was 6, while unphosphorylated chains predominated at lengths of 5-7 for liver, and 6-8 for lung. Only for frontal cortex unphosphorylated and phosphorylated polymers showed a similar distribution. This should be correctly mentioned in the text.

Figure 5: The authors mention that Ewing sarcoma most likely derives from connective tissue near the bone. They say that glycogen abundance in Ewing sarcoma was comparable to adjacent skeletal muscle (Fig. 5F). Was phosphorylation pattern in skeletal muscle also the same? And what about bone marrow mesenchymal stem cells, which have been discussed as most likely candidate cell-type of origin for Ewing sarcoma? Glycogen abundance, architecture and phosphorylation in MSC should also be tested and compared to Ewing sarcoma.

Figure 6E: Demonstration of successful GYS1 knockout by Western blot should be included into the figure. Also, does GYS1 knockout affect growth characteristics of A673 cells in vitro? This should be shown. If there was already an effect on growth or viability of the cells in monolayer culture, this would help explain the drastic in vivo effect.

Fig 6G-H: IC50 value for Guaiacol in monolayer culture is pretty high (20uM). What about spheroid cultures, which are known to be closer to tumors and much less resistant to many treatments than are 2D cultures?

Fig 6I: As glycogen synthesis is playing important metabolic roles in many organs, was there any and which type of toxicity seen in treated mice? These data should be provided as a supplement.

Fig 6: The text says that "compared to normal tissues, ES glycogen exhibits marked increases in total phosphate content". Please, include analysis of skeletal muscle (which is mentioned in line 81 but not shown), adipose tissue and MSC among

normal tissues here (also see comment above). It is not clear, what "normal tissue" in Fig 6K-L is referring to. It is not surprising that normal bone tissue, which the authors showed to largely lack glycogen deposits, are also low for phosphorylated glycogen polymers.

Fig 6I, 6P and Fig S6G: The in vivo data lack an indication of the numbers of mice and tumors included per group.

Fig 6M: The Kaplan Meier curve lacks an indication of numbers of patients with high and low EPM2A (laforin) expression. As laforin in Ewing sarcoma is generally low, and it appears that the number of patients with even lower laforin levels is small, the data need to be confirmed on an independent data set (e.g. GSE63157 or other).

How does laforin expression in Ewing sarcoma compare to MSC, the likely tissue of origin, or skeletal muscle, which shows similar glycogen levels as Ewing sarcoma?

Fig 6P and S6G both show tumor formation with laforin overexpression in A673. What is the difference? Should not one of them be TC32?

Figure 6O: What is the normal tissue in this figure?

Minor:
On page 5, line 37, authors mention an incidence of Ewing sarcoma of ~10,000 children, adolescents, and young adults each year. To which population does this number refer to, US or whole world?

Page 6, line 67: What does 25mM DMEM mean? This sentence should be rephrased.

Referee #3 (Remarks for Author):

The manuscript by Young et al. describes a method to quantify cellular glycogen contents and qualities in situ with spacial resolution. Using this method, the authors compare a wide range of tumor entitles and found that especially Ewing sarcoma (EwS) tumors contain a high amount of hyperphosphorylated cytoplasmic glycogen. Blocking glycogen synthetase by knockout or a small molecule inhibitor depleted EwS cells from glycogen, which blocked tumor growth in vivo.
This study is well written, novel and of great interest, and highlights EwS as a cancer entity in which blockage of glycogen storage may be of therapeutic use. However, there are a few issues that need to be addressed before publication:

1. The survival data shown in Figure 6M is likely wrong since the Savola dataset contains only a few EwS cases. The dataset GSE 17679 comprises 117 samples out of which 18 are normal tissues (skeletal muscle) and 11 EwS cell lines. The remaining 88 patient samples are duplicate uploads of 44 "true" samples. Hence, the true number of cases available for Kaplan-Meier survival analyses is 44 and not 117 as reported by the authors. This error must be corrected before publication. There are other survival data in the literature (e.g. Musa et al. 2019) that may be better suited.

2. The fact that squamous cell lung carcinoma contains less glycogen than lung adenocarcinoma is trivial and should not be reported here as a novelty. In fact, every pathologist can see this by H&E stains, and validate by PAS stains. The data reported here are correct, but the authors should "sell" this point rather as a control for their method than as a novel finding.

3. The data on EwS growth inhibition by GSY1 KO and pharmacological inhibition are very interesting. Since in the experiment of GYSi also tumors in the treatment group are available. The authors should compare those tumors to the controls by histology. Are there less mitoses? Is there any sign of differentiation? Are there differences in apoptosis/necrosis?

4. The reference by Riggi et al is debated. In fact, two alerting letters to the editor accompany this review that is in many aspects not fully accurate. The authors are advised to not use this reference that is viewed critically in the field.

5. It would be very interesting to show whether EwS metastases exhibit different levels of (hyperphosphorylated) glycogen compared to primary tumors.

6. The authors should explore whether important genes involved in glycogen metabolism (such as glycogen synthetase or laforin) are impacted by the pathognomonic gene fusion in EwS. There are comprehensive datasets available to carry out such analyses (e.g. GSE176339 for genomics; Orth et al. 2021 bioRxiv for proteomics). It would be very interesting to show whether these genes are targets of the fusion, which may explain the overabundance of glycogen in EwS.

We would like to thank the reviewers for their enthusiasm regarding the significance of our manuscript as well as their constructive criticisms. We have carefully considered each comment and addressed <u>all points</u> with additional data, discussions, and references to improve the overall quality of the manuscript. Reviewer's comments are in grey and we respond to reviewer's comments in black.

Referee #1 (Comments on Novelty/Model System for Author):

The manuscript concerns the analysis of glycogen using imaging mass spectrometry. The authors report higher levels of glycogen in a number of tumors, of which the highest levels were found in Ewing's Sarcoma. It is then reported that interfering with glycogen synthase, the enzyme that builds glycogen, via CRISPR-Cas9 knockouts or using the inhibitor guaiacol, reduces tumor growth in Ewing Sarcoma. This Ewing Sarcoma part is by far the strongest part of the work, and clearly the approach could be applied to many other diseases to investigate the potential role of glycogen in their development. My main concern is the very observational nature of the data, and the fact that the numbers of subjects are not provided and the statistical analysis is far from clear, or far from satisfactory. For example. Figure 2C, the statistical test (one way ANOVA) were applied using pixel data from one imaging mass spectrometry dataset. It is not appropriate to use such tests on pixel data because the data is not independent...

We appreciate the reviewer's positive comments regarding our work and have performed additional experiments and revised the manuscript based on the reviewer's suggestions. Please find our responses to concerns point-by-point below.

Referee #1 (Remarks for Author):

1). The manuscript concerns the analysis of glycogen using imaging mass spectrometry. The authors report higher levels of glycogen in a number of tumors, of which the highest levels were found in Ewing's Sarcoma. It is then reported that interfering with glycogen synthase, the enzyme that builds glycogen, via CRISPR-Cas9 knockouts or using the inhibitor guaiacol, reduces tumor growth in Ewing Sarcoma. This Ewing Sarcoma part is by far the strongest part of the work, and clearly the approach could be applied to many other diseases to investigate the potential role of glycogen in their development. My main concern is the very observational nature of the data, and the fact that the numbers of subjects are not provided and the statistical analysis is far from clear, or far from satisfactory. For example, in Figure 2C, the statistical test (one way ANOVA) were applied using pixel data from one imaging mass spectrometry dataset. It is not appropriate to use such tests on pixel data because the data is not independent...

In the revised manuscript, we have improved on the data analysis aspect of the manuscript based on reviewer suggestions, implementing additional statistical tests instead of relying observational data. We apologize for the less than clear numbers of subjects and have added details for n numbers and statistical testing in figure legends and the method section. For figure 2C, we performed multiple comparison tests that account for more than 1 dependent variables. We have updated this in the method section.

The manuscript is also currently too observational. Instead of many paired H&E and glycogen images, but in which any histological annotations have not been mapped onto the mass spectrometry images, it would be much more objective to extract the average signatures from specific histopathological regions, for multiple patient samples, and report the average levels in these regions, with appropriate statistical tests. i.e. please provide quantifiable data.

We agree with the reviewer regarding the optimal methodology to analyze these data. We have extracted average signatures from specific histopathological regions from all patients and report the chain length distribution for all regions followed by ANOVA and multiple comparison test. The data are included in Supplemental Figure 5 and updated text in page 6 and glycogen chain length analysis can be found below.

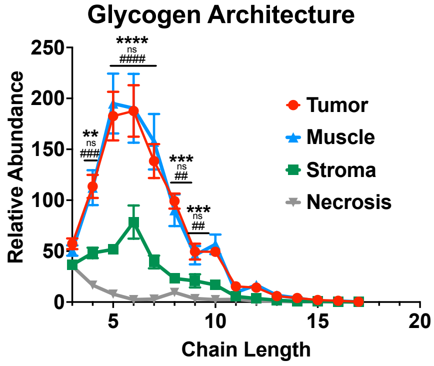

The section on simultaneous N-linked glycan and glycogen should not be included here, as it serves little purpose (never referred to later) and the authors did not demonstrate if/how it affects sensitivity. Using two enzymes will release both molecular types, but the added complexity of the molecular information is expected to lead to competition for charge (during ionization) and capacity if using ion trapping technologies (e.g. FTICR). Here the novelty is on glycogen imaging. This reviewer advises the authors to focus better, and develop a separate analytical investigation of this dual enzyme approach in which sensitivity is properly established.

We appreciate the reviewer’s comments and therefore removed FTICR data set from Supplemental Figure 1. Instead, we performed the cross-institution validation using the Bruker TIMS-TOF Flex instrument with time-of-flight detector to demonstrate that this method yields robust results on multiple platforms, which will be key moving forward for this new method. New data are included in Supplemental Figure 1.

Please improve figures, particularly Figure 1 (many screen grabs of insufficient resolution, and red and blue channels of 1G are not legible). Currently many panels in Figure 1 are barely comprehensible.

We have improved the image quality in Figure 1and we hope high-resolution images are clearly visible now. Further, there is a size limit for figures during the manuscript submission process that require compression, uncompressed images files will be submitted with the final accepted manuscript.

2). There is some evidence of publication bias in this manuscript. Referring to figure 1B on page 4 the authors describe "a gradient of glycogen stores radiating away from the central vein". This gradient is far from clear, and should be explicitly demonstrated by extracting the gradient data from the imaging mass spectrometry datasets, for multiple mouse liver samples (from different animals).

We thank the reviewer for bringing this concern to our attention. Based on this comment, we performed additional analyses by quantifying each pixel from the central vein to Glisson’s capsule to show a reduction of glycogen from the central vein. These data are added as Supplemental Figure 2 and presented below.

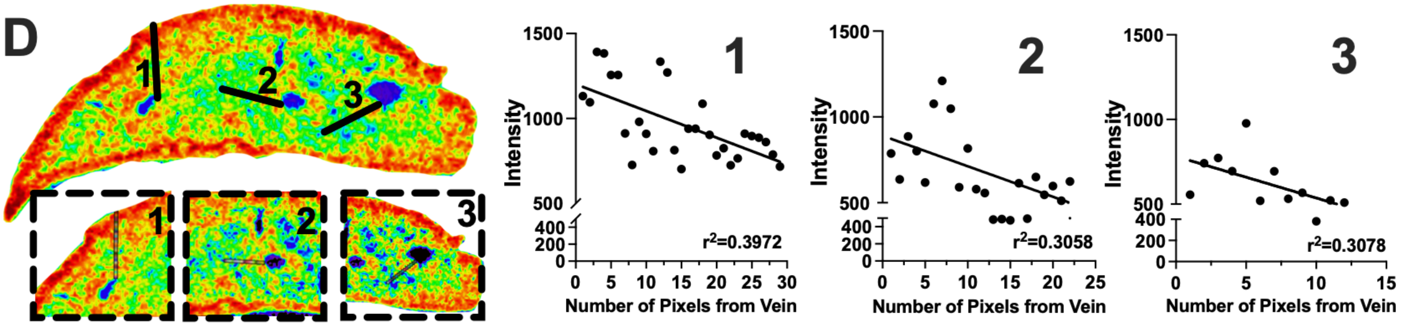

Similarly in Figure 6A the the authors describe on page 6 the "normal bone tissue, glycogen predominantly accumulated in the bone marrow with less extent in the outer calcified bone". However the methodology is based on enzymatic activity of the isomyalase and the authors do not appear to have ever considered if the enzyme has equal access to all tissue types - in this instance enzyme is much less likely to access to glycogen encolsed in the bone... some critical analysis is lacking.

Regarding text describing the bone sample, we would like to apologize for the errors in the original text. The bone sample utilized in Figure 6A is indeed decalcified bone, a standard procedure performed by the Biospecimen Procurement and Translational Pathology Facility at the Markey Cancer Center. We have updated this point both in the main text and the method section accordingly.

This reviewer is postive about the work, but the results need to be presented in a more quantitative manner, and the statistics more clearly described, especially the numbers of biological samples from which the conclusions were drawn.

We appreciate the reviewer’s comments to improve the manuscript. We have expanded our statistical section in the methods, and figure captions as well as added numbers of samples to figure captions.

Minor.
4). Figure 5C - the label M in the figure should be N (for Necrosis)
We thank the reviewer for catching this error. The M is abbreviation for muscle and was sightly shifted to the left. This placement has now been corrected to reflect the muscle region adjacent to the tumor. We have updated Figure 5 and the figure legend respectively, updated image can be found below.

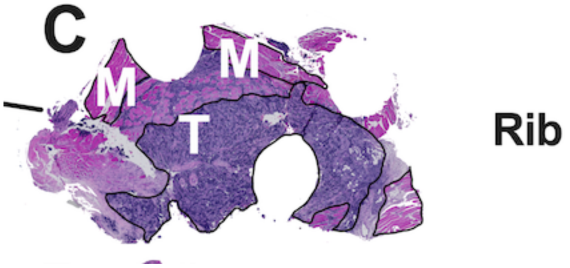

5) Figure 6B. Please define what high, outer, and low refer too as it is never defined.

We would like to thank the reviewer for point this issue out. We have corrected the labeled that corresponding to bone, connective tissue, and bone marrow. This is updated in the revised version of the figures and text.

6) Figure legends. Please provide legends that explain symbols used within the figures, so as to spare the reader from trawling through lengthy captions.
page 6.
We have added key and corresponding explanations as part of figure panels in all figures.

7) Please define what is meant by GSD,,,.
GSD is the abbreviation of glycogen storage disease. We have made the appropriate change in the text of the manuscript.

Referee #2 (Comments on Novelty/Model System for Author):

I am not an expert in MALDI-MSI and therefore cannot really judge the technical quality of the corresponding experiments in detail. However, the demonstration of the versatility of the introduced technological pipeline to assess glycogen content, spatial distribution and architecture on FFPE slides of various tissues is convincing and the results are novel laying the basis for future functional studies. They also identify a new avenue to target Ewing sarcoma and may therefore impact patient treatment with an innovative approach. However, potential toxicity issues arising from treatment with glycogen biosynthesis targeting compounds are not addressed.

We appreciate the reviewer's positive comments regarding our work. We have performed additional experimental validation in line with reviewer's comments to improve the manuscript. Please find our point-by-point response to concerns below.

Referee #2 (Remarks for Author):

Glycogen is a key intracellular glucose metabolite involved in a still expanding number of physiological processes. Our knowledge on tissue and cell-type specific glycogen stores has so far largely been limited to its detectability by periodic acid-Schiff (PAS) reaction. In this manuscript, Young et al. utilize a novel matrix-assisted laser desorption/ionization mass spectrometry-based imaging pipeline for the analysis of FFPE sections to describe architectural and spatial heterogeneity in glycogen expression of normal mouse and human tissues, and to characterize quantitative and qualitative glycogen patterns in some cancers with a focus on Ewing sarcoma, a pediatric bone tumor characterized by high PAS positivity. For the first time, they report the spatial distribution of glycogen chains of distinct lengths, architecture and phosphate content with high resolution in tissues and cell types of major organs, prostate cancer, non-small cell lung cancer and Ewing sarcoma. Importantly, by genetic (CRISPR/Cas9) and pharmacologic knockout/knockdown of glycogen synthetase GYS1 or ectopically forcing expression of glycogen phosphatase laforin, they provide evidence that targeting the glycogen biosynthetic pathway significantly reduces Ewing sarcoma growth in vivo. These findings may open new therapeutic avenues for the treatment of this devastating disease.

Specific comments:
Figure S1C: To illustrate robustness of the method across different MALDI-MSI platforms, Figure S1C presents MALDI-MSI spatial distribution of glycogen in a rat brain using a Bruker FTICR mass spectrometer, and text says that "a similar chain length distribution was observed". Similar to what? To the results obtained with the Waters Synapt G2 XS ion mobility-enabled mass spectrometer? These are not shown for mouse brain. Or to mouse liver, which is discussed in the preceding lines?

We agree with the reviewer that same mouse tissue should be used for cross institution validation of the methodology on the two instruments. Therefore, we analyzed an adjacent section of the same mouse liver tissue used in figure 1 using the new Bruker TIMS-TOF flex instrument at MUSC. The TIMS-TOF is also a time-of-flight system similar to the Waters G2. Adjacent mouse tissue sections exhibit similar glycogen polymer chain length pattern on each instrument. Importantly, these samples were analyzed on a Waters instrument at U. of Kentucky and a Bruker instrument at MUSC. We have updated the figure accordingly and representative figure can be found below.

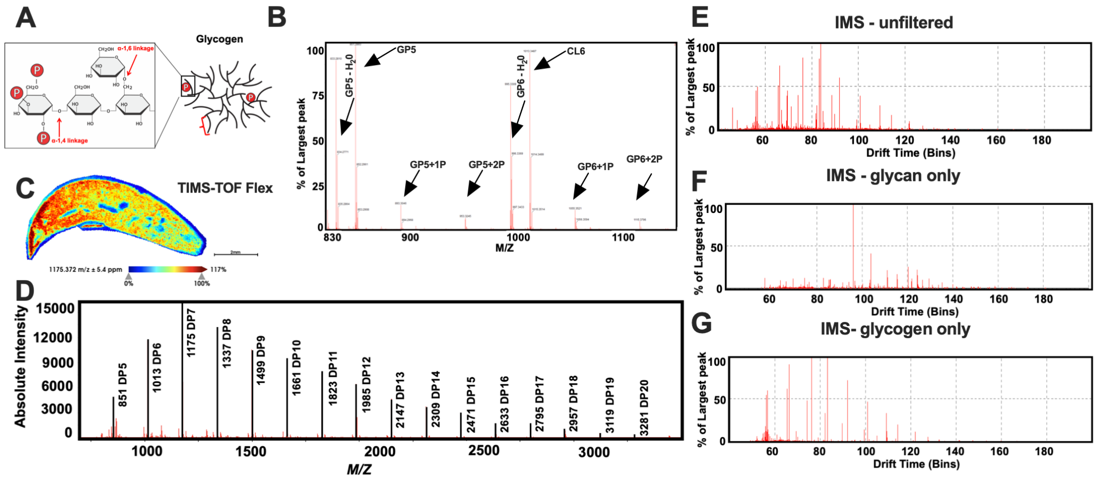

Figure S3C, S3F, S3I: Text says that phosphorylated chain length in non-diseased tissues displayed similar polymeric distribution

compared to non-phosphorylated glycogen species. However, it appears that for liver and lung, abundance of phosphorylated polymers was highest at a chain length of around 4, and for brain frontal cortex it was 6, while unphosphorylated chains predominated at lengths of 5-7 for liver, and 6-8 for lung. Only for frontal cortex unphosphorylated and phosphorylated polymers showed a similar distribution. This should be correctly mentioned in the text

We appreciate the authors for bringing this to our attention. The text now states: “Phosphorylated chain length displayed similar polymeric distribution compared to the non-phosphorylated species glycogen with unique differences among highest phosphorylated glycogen chain length. For examples, for lung and liver, phosphorylated polymers was highest at a chain length of 3 and 4 respectively, while unphosphorylated chains shown highest abundance at lengths of 5-7 for liver, and 6-8 for lung. (Fig. S3C, S3F and S3I).”

Figure 5: The authors mention that Ewing sarcoma most likely derives from connective tissue near the bone. They say that glycogen abundance in Ewing sarcoma was comparable to adjacent skeletal muscle (Fig. 5F). Was phosphorylation pattern in skeletal muscle also the same?

Based on the reviewer’s comments, we performed additional analysis of glycogen phosphate between tumor (T) and muscle (M) between two different patients and found no difference between the two regions. These new data are incorporated in Supplemental Figure 6 and can be found below.

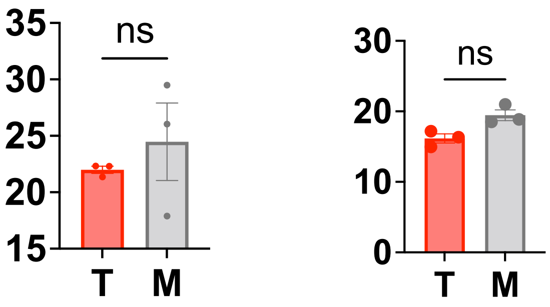

And what about bone marrow mesenchymal stem cells, which have been discussed as most likely candidate cell-type of origin for Ewing sarcoma? Glycogen abundance, architecture, and phosphorylation in MSC should also be tested and compared to Ewing sarcoma.

Our MALDI workflow does not currently have the resolution to detect mesenchymal stem cells. Therefore, to answer the reviewer’s question, we purchased bone marrow-derived mesenchymal stem cells and analyzed glycogen abundance, architecture, and phosphate content and compared to the human derived A673 model Ewing cell line[1-4]. These data are incorporated in Supplemental Figure 5. There was no significant difference between the Ewing sarcoma cell line and A673 and mesenchymal stem cells, data shown below.

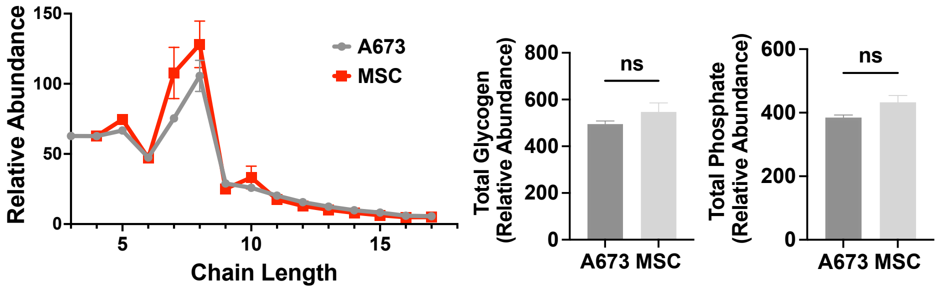

Based on the additional mesenchymal stem cell data, we have revised the discussion and speculate that based on the similar glycogen content of muscle, mesenchymal stem cell and tumors, additional work is warranted to study the connection and cellular origins of Ewing’s Sarcoma.

Figure 6E: Demonstration of successful GYS1 knockout by Western blot should be included into the figure. Also, does GYS1 knockout affect growth characteristics of A673 cells in vitro? This should be shown. If there was already an effect on growth or viability of the cells in monolayer culture, this would help explain the drastic in vivo effect.

We have included the western blot showing successful knockout of GYS1 and incorporated in Fig. 6F. Based on the reviewer’s question, we also performed an *in vitro* cell viability assay that demonstrates no inherent growth defect in cells with GYS1 knockout. The *in vitro* conditions likely do not replicate the Ewing’s tumor microenvironment due to high levels of glucose and 21% oxygen, but we agree this is an important control. These data are included in Figure 6 with the xenograft study, Supplemental Figure 6, and are presented below.

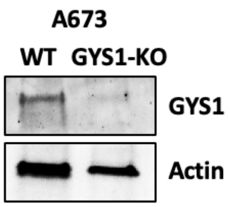

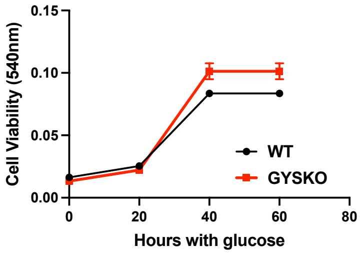

Fig 6G-H: IC50 value for Guaiacol in monolayer culture is pretty high (20uM). What about spheroid cultures, which are known to be closer to tumors and much less resistant to many treatments than are 2D cultures?

We appreciate the reviewer's comment about the IC50 of Guaiacol. This small molecule inhibitor has been shown to be effective in Adult Polyglucosan Body Disease (APBD) where the authors utilized a similar elevated EC50[5]. Based on this literature, we performed the assay under similar conditions to identify the 20uM concentration.

Fig 6I: As glycogen synthesis is playing important metabolic roles in many organs, was there any and which type of toxicity seen in treated mice? These data should be provided as a supplement.

Glycogen is synthesized by Gys1 in all other tissues except the liver and we targeted Gys1 in this study. The reviewer is correct that glycogen synthesis plays important roles in many organs. However, multiple groups have demonstrated that $\geq$50% reduction in glycogen levels does not result in health consequences in mouse models or in humans [6-9] .

In this study, mice were carefully monitored through coat color, alertness, and general mobility and we did not observe any adverse effects of GYSi in our cohort of mice. Similar findings have also been reported for glycogen storage disease in humans, as almost all GSDs are caused by the inability to utilize glycogen or complete ablation of glycogen synthesis, rather than partial GYS deficiencies[10]. Further, GSDs usually take 1 to several decade of life to show symptoms.

In 2009, a highly potent glycogen phosphorylase inhibitor GSK1362885 was orally administrated for 7 days in 23 participants during a phase 1 clinical trial (Clinical Trial identifier: NCT01013766). The study concluded showing no measurable side effects from oral administration of GSK1362885. As glycogen phosphorylase releases glucose-1-phosphate, this inhibitor reduces glycogen utilization.

Additionally, the *PPP1R3A* gene encodes a positive regulator of glycogen synthesis. A truncating mutation was identified in *PPP1R3A* that results in ~65% reduction of glycogen levels and this mutation is found in ~1.1% of Europeans[11]. Introducing the same mutation into mice also resulted in an ~65% reduction in glycogen synthesis and no observed cardiac or other phenotype was identified in the mice. A recent study investigated the consequences of this genetic mutation in humans using UK Biobank data[12]. They identified no association between *PPP1R3A* truncation mutations and cardiac parameters, including ventricular ejection fraction, wall thickness, maximum heart rate, and maximum workload. They also observed no changes in serum glucose or serum metabolites. Importantly, there was no association between *PPP1R3A* truncation mutations and key health outcomes. These data strongly suggest that at least 65% reduction in glycogen synthesis is well tolerated. Given these data, we feel it is not surprising that the mice in our study did not present with any adverse symptoms.

Fig 6: The text says that "compared to normal tissues, ES glycogen exhibits marked increases in total phosphate content". Please, include analysis of skeletal muscle (which is mentioned in line 81 but not shown), adipose tissue and MSC among normal tissues here (also see comment above). It is not clear, what "normal tissue" in Fig 6K-L is referring to. It is not surprising that normal bone tissue, which the authors showed to largely lack glycogen deposits, are also low for phosphorylated glycogen polymers.

The "normal" referred to the bone marrow, this is corrected in the figure, text, and figure legends. Per this comment, we now include the analysis of tumor glycogen and glycogen phosphate among other tissues in the available Ewing tumor specimens (mesenchymal stem cells were not included in this analysis due to *in vitro* vs. *in vivo* effects). Three technical replicates/regions of interests were collected from each region of all specimens. These data are included in Supplemental Figure 6 and presented below.

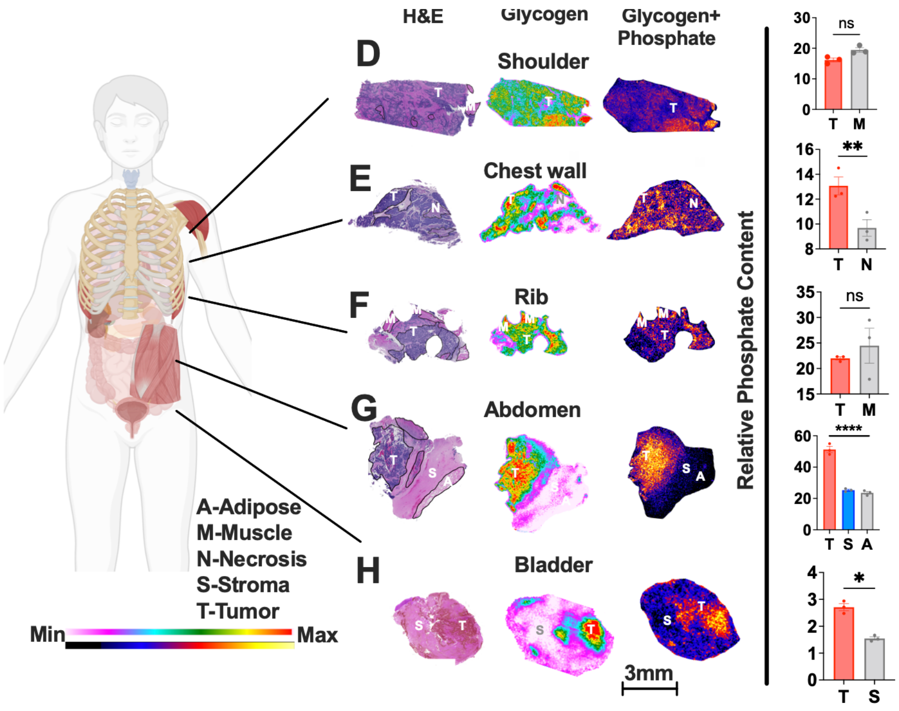

Fig 6I, 6P and Fig S6G: The in vivo data lack an indication of the numbers of mice and tumors included per group.

The figure captions have been edited to include the number of mice per group (n=8).

Fig 6M: The Kaplan Meier curve lacks an indication of numbers of patients with high and low EPM2A (laforin) expression. As laforin in Ewing sarcoma is generally low, and it appears that the number of patients with even lower laforin levels is small, the data need to be confirmed on an independent data set (e.g. GSE63157 or other). Check p value for KP.

We would like to thank the reviewer for this point and acknowledge that the number is small. Therefore, we have removed the Kaplan Meier curve and replaced it with laforin expression from three independent data sets. In agreement with our hypothesis, laforin is significantly downregulated in all three data sets in the tumors. Using the independent data sets of GSE17679 Ewing sarcoma (n=65) and normal muscle samples (n=18), GEOD-17674 Ewing sarcoma (n=44) and normal muscle (n=18) and GEOD-37371 Ewing sarcoma (n=20) we confirmed lower laforin expression in all three datasets as compared to normal muscle. These data were incorporated into Figure 6 and are below.

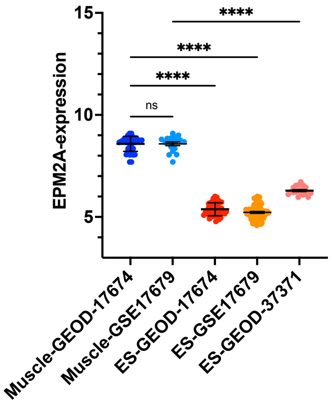

How does laforin expression in Ewing sarcoma compare to MSC, the likely tissue of origin, or skeletal muscle, which shows similar glycogen levels as Ewing sarcoma?

Using bone-marrow derived mesenchymal stem cells, we performed a western blot for laforin and we were unable to detect laforin by western blot in the MSCs, suggesting alternative epigenetic programming in MSCs that require future studies. These data are included in Supplemental Figure 5 and presented below.

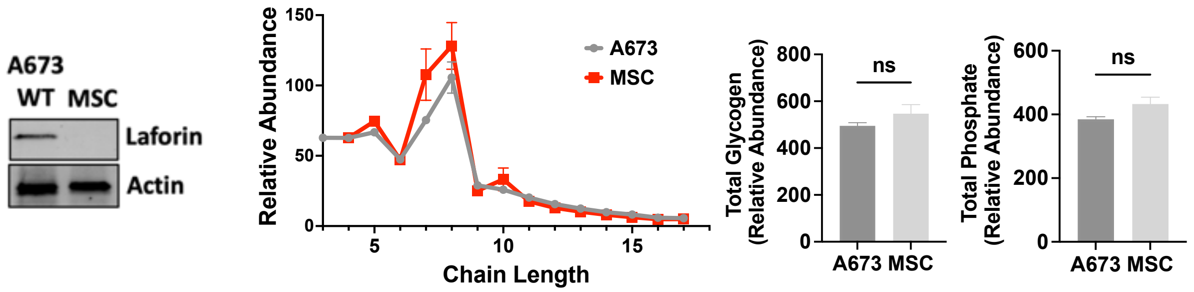

Fig 6P and S6G both show tumor formation with laforin overexpression in A673. What is the difference? Should not one of them be TC32?

We appreciate the reviewer for finding this error. Indeed, S6G should be TC32. We have corrected this typographical error.

Figure 6O: What is the normal tissue in this figure?

We have renamed this tissue in the figure to improve clarity. The normal tissue observed in 6O is muscle.

Minor:
On page 5, line 37, authors mention an incidence of Ewing sarcoma of ~10,000 children, adolescents, and young adults each year. To which population does this number refer to, US or whole world?
We have changed the text to reflect both the US and world populations of ewing sarcoma patients. Line 240: ES is a pediatric bone and soft tissue malignancy affecting 2.93 children per 1,000,000. Approximately 250 children and adolescents are diagnosed each year in the United States.[13,14]"

Page 6, line 67: What does 25mM DMEM mean? This sentence should be rephrased.
25mM DMEM refers to DMEM cell culture media with 25mM glucose. We have rephrased this sentence as "cells were grown to 80% confluency in standard cell culture conditions for ES cells." 25mM glucose DMEM has been moved to the method section.

Referee #3 (Remarks for Author):

The manuscript by Young et al. describes a method to quantify cellular glycogen contents and qualities in situ with spacial resolution. Using this method, the authors compare a wide range of tumor entitles and found that especially Ewing sarcoma (EwS) tumors contain a high amount of hyperphosphorylated cytoplasmic glycogen. Blocking glycogen synthetase by knockout or a small molecule inhibitor depleted EwS cells from glycogen, which blocked tumor growth in vivo.
This study is well written, novel and of great interest, and highlights EwS as a cancer entity in which blockage of glycogen storage may be of therapeutic use. However, there are a few issues that need to be addressed before publication:

We appreciate the reviewer's positive comments regarding our work. We performed additional experiments and analyses based on the reviewer's suggestions to improve the manuscript. Please find our response to concerns point-by-point below.

1. The survival data shown in Figure 6M is likely wrong since the Savola dataset contains only a few EwS cases. The dataset GSE 17679 comprises 117 samples out of which 18 are normal tissues (skeletal muscle) and 11 EwS cell lines. The remaining 88 patient samples are duplicate uploads of 44 "true" samples. Hence, the true number of cases available for Kaplan-Meier survival analyses is 44 and not 117 as reported by the authors. This error must be corrected before publication. There are other survival data in the literature (e.g. Musa et al. 2019) that may be better suited.

We would like to thank the reviewer for this point and acknowledge that the number is small. Therefore, we have removed the Kaplan Meier curve and replaced it with laforin expression from three independent data sets. In agreement with our hypothesis, laforin is significantly downregulated in all three data sets in the tumors. Using the independent data sets of GSE17679 Ewing sarcoma (n=65) and normal muscle samples (n=18), GEOD-17674 Ewing sarcoma (n=44) and normal muscle (n=18) and GEOD-37371 Ewing sarcoma (n=20) we confirmed lower laforin expression in all three datasets as compared to normal muscle. These data are incorporated into Figure 6 and are below.

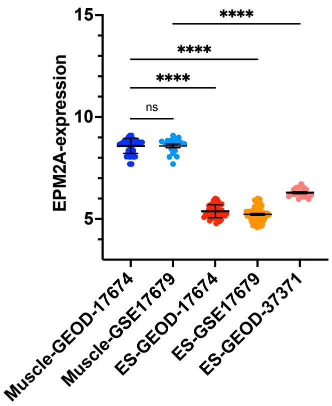

2. The fact that squamous cell lung carcinoma contains less glycogen than lung adenocarcinoma is trivial and should not be reported here as a novelty. In fact, every pathologist can see this by H&E stains, and validate by PAS stains. The data reported here are correct, but the authors should "sell" this point rather as a control for their method than as a novel finding.

Per the reviewer's suggestion, we have edited this point in the main text of the manuscript. See revised text below.
"As expected, intratumoral glycogen was observed in LUAD and not in LUSC (Fig. 4A-C). Interestingly, glycogen was identified in stroma, blood vessel walls, and residual cartilage in LUSC (Fig. 4A-B). Collectively, these results highlight the need for spatial glycogen analysis as pooled LUSC and prostate samples would likely be false positive for intratumoral glycogen accumulation based on glycogen in non-tumor regions."

3. The data on EwS growth inhibition by GSY1 KO and pharmacological inhibition are very interesting. Since in the experiment of GYSi also tumors in the treatment group are available. The authors should compare those tumors to the controls by histology. Are there less mitoses? Is there any sign of differentiation? Are there differences in apoptosis/necrosis?

Based on the reviewer's suggestions, we performed immunohistochemistry for cleaved caspase 3 (apoptosis) and Ki67 (mitotic index). Using the HALO digital pathology suite that offers unbiased quantitation of the entire slide within the defined tumor regions, we observed a very modest increase in the number of CC3 cells in the guaiacol treated tumors (P=0.054). Interestingly, Ki67 staining displayed significantly reduced mitotic index within the tumor regions of guaiacol treated tumors. These data suggest guaiacol exhibits anti-proliferative effect rather than cytotoxic effects in the xenograft model of Ewing tumors. These data have been added to Supplemental Figure 6 and we have revised the text. Figures can be found below.

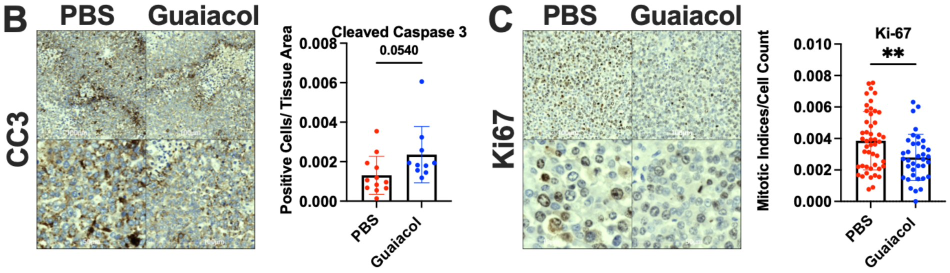

4. The reference by Riggi et al is debated. In fact, two alerting letters to the editor accompany this review that is in many aspects not fully accurate. The authors are advised to not use this reference that is viewed critically in the field.
We have added an additional Ewing sarcoma review (Grunewald et al) to which we believe captures additional ES aspects and expands on the ES literature to provide readers with a solid foundation of past work and current hypotheses.

5. It would be very interesting to show whether EwS metastases exhibit different levels of (hyperphosphorylated) glycogen compared to primary tumors.

We appreciate the interest of the reviewer in the effect of glycogen on EwS metastasis. While we agree it would be interesting to analyze EwS metastases, it has been incredibility difficult to obtain matched primary and metastatic Ewing tumors. We are working with surgeons to collect paired tissues in hopes of publishing these findings in a future follow-up manuscript.

6. The authors should explore whether important genes involved in glycogen metabolism (such as glycogen synthetase or laforin) are impacted by the pathognomonic gene fusion in EwS. There are comprehensive datasets available to carry out such analyses (e.g. GSE176339 for genomics; Orth et al. 2021 bioRxiv for proteomics). It would be very interesting to show whether these genes are targets of the fusion, which may explain the overabundance of glycogen in EwS.

We would like to thank the reviewer for this suggestion. Since EwS glycogen exhibits structural similarities with mesenchymal stem cells and adjacent muscle tissue, we hypothesize that the glycogen phenotype is retained through cell lineages of EwS. This certainly does not preclude the effects of fusion protein on glycogen metabolic enzymes, we will explore this possibility in future studies through rigorous molecular and cellular means and report these in a future publication.

1 Lin, P. P., Wang, Y. & Lozano, G. Mesenchymal stem cells and the origin of Ewing's sarcoma. *Sarcoma* **2011** (2010).

2 Riggi, N. *et al.* EWS-FLI-1 expression triggers a Ewing's sarcoma initiation program in primary human mesenchymal stem cells. *Cancer Res* **68**, 2176-2185, doi:10.1158/0008-5472.CAN-07-1761 (2008).

3 Gonzalez, I., Vicent, S., de Alava, E. & Lecanda, F. EWS/FLI-1 oncoprotein subtypes impose different requirements for transformation and metastatic activity in a murine model. *J Mol Med (Berl)* **85**, 1015-1029, doi:10.1007/s00109-007-0202-5 (2007).

4 Riggi, N. *et al.* Development of Ewing's sarcoma from primary bone marrow-derived mesenchymal progenitor cells. *Cancer Res* **65**, 11459-11468, doi:10.1158/0008-5472.CAN-05-1696 (2005).

5 Kakhlon, O. *et al.* Guaiacol as a drug candidate for treating adult polyglucosan body disease. *JCI Insight* **3**, doi:10.1172/jci.insight.99694 (2018).

6 Nitschke, S. *et al.* An inducible glycogen synthase-1 knockout halts but does not reverse Lafora disease progression in mice. *Journal of Biological Chemistry* **296** (2021).

7 Pederson, B. A. *et al.* Glucose Metabolism in Mice Lacking Muscle Glycogen Synthase. *Diabetes* **54**, 3466-3473, doi:10.2337/diabetes.54.12.3466 (2005).

8 Duran, J., Saez, I., Gruart, A., Guinovart, J. J. & Delgado-García, J. M. Impairment in long-term memory formation and learning-dependent synaptic plasticity in mice lacking glycogen synthase in the brain. *Journal of Cerebral Blood Flow & Metabolism* **33**, 550-556 (2013).

9 Duran, J. *et al.* Astrocytic glycogen accumulation drives the pathophysiology of neurodegeneration in Lafora disease. *Brain* **144**, 2349-2360 (2021).

10 Wolfsdorf, J. I. & Weinstein, D. A. Glycogen storage diseases. *Reviews in Endocrine and Metabolic Disorders* **4**, 95-102 (2003).

11 Savage, D. B. *et al.* A prevalent variant in PPP1R3A impairs glycogen synthesis and reduces muscle glycogen content in humans and mice. *PLoS medicine* **5**, e27 (2008).

12 Homburgeer, J. R. *et al.* Genetic reduction of muscle glycogen is well tolerated in UK Biobank participants. (2021).

13 Grunewald, T. G. P. *et al.* Ewing sarcoma. *Nat Rev Dis Primers* **4**, 5, doi:10.1038/s41572-018-0003-x (2018).

14 Society, A. American Cancer Society. Cancer Facts & Figures 2020. *Am Cancer Soc*, 1-52 (2020).

21st Jul 2022

Dear Dr. Sun,

Thank you for the submission of your revised manuscript to EMBO Molecular Medicine. I am pleased to inform you that we will be able to accept your manuscript pending the following final amendments:

1) Figures: We noticed that you use the same representative image between figures. As suggested before, please state in the figure legends that the image is reused.
2) In the main manuscript file, please do the following:
- Remove all figures from the main manuscript file and leave only figure legends.
- In M&M, add statistical paragraph that should reflect all information that you have filled in the Authors Checklist, especially regarding randomization, blinding, replication.
- In M&M, please include statement that the experiments using human samples conformed to the principles set out in the WMA Declaration of Helsinki and the Department of Health and Human Services Belmont Report. Please also amend it in the "Author Checklist" as well.
- Please rename "Conflict of Interest" to "Disclosure Statement & Competing Interests". We updated our journal's competing interests policy in January 2022 and request authors to consider both actual and perceived competing interests. Please review the policy https://www.embopress.org/competing-interests and update your competing interests if necessary.
3) Author contributions: Please specify author contributions in our submission system. CRediT has replaced the traditional author contributions section because it offers a systematic machine readable author contributions format that allows for more effective research assessment. You are encouraged to use the free text boxes beneath each contributing author's name to add specific details on the author's contribution. More information is available in our guide to authors: https://www.embopress.org/page/journal/17574684/authorguide#authorshipguidelines
4) Synopsis: Every published paper now includes a 'Synopsis' to further enhance discoverability. Synopses are displayed on the journal webpage and are freely accessible to all readers. They include separate synopsis image and synopsis text.
- Synopsis image: Please provide a striking image or visual abstract as a high-resolution jpeg file 550 px-wide x (250-400)-px high to illustrate your article.
- Synopsis text: Please provide a short standfirst (maximum of 300 characters, including space) as well as 2-5 one sentence bullet points that summarise the paper as a .doc file. Please write the bullet points to summarise the key NEW findings. They should be designed to be complementary to the abstract - i.e. not repeat the same text. We encourage inclusion of key acronyms and quantitative information (maximum of 30 words / bullet point). Please use the passive voice.
- Please check your synopsis text and image before submission with your revised manuscript. Please be aware that in the proof stage minor corrections only are allowed (e.g., typos).
5) For more information: Please remove corresponding author's e-mail address. This space should be used to list relevant web links for further consultation by our readers. Could you identify some relevant ones and provide such information as well? Some examples are patient associations, relevant databases, OMIM/proteins/genes links, author's websites, etc...
6) Source data: We encourage you to include the source data for figure panels that show essential data. Numerical data should be provided as individual .xls or .csv files (including a tab describing the data). For blots or microscopy, uncropped images should be submitted (using a zip archive if multiple images need to be supplied for one panel). Please check "Author Guidelines" for more information. https://www.embopress.org/page/journal/17574684/authorguide#sourcedata
7) As part of the EMBO Publications transparent editorial process initiative (see our Editorial at http://embomolmed.embopress.org/content/2/9/329), EMBO Molecular Medicine will publish online a Review Process File (RPF) to accompany accepted manuscripts. This file will be published in conjunction with your paper and will include the anonymous referee reports, your point-by-point response and all pertinent correspondence relating to the manuscript. Let us know whether you agree with the publication of the RPF and as here, if you want to remove or not any figures from it prior to publication. Please note that the Authors checklist will be published at the end of the RPF.
8) Please provide a point-by-point letter INCLUDING my comments as well as the reviewer's reports and your detailed responses (as Word file).

I look forward to reading a new revised version of your manuscript as soon as possible.

Yours sincerely,

Zeljko Durdevic

Zeljko Durdevic
Editor
EMBO Molecular Medicine

***** Reviewer's comments *****

Referee #2 (Remarks for Author):

The authors adequately addressed all points raised by this reviewer.

Referee #3 (Remarks for Author):

The authors addressed all my concerns

The authors performed the requested editorial changes.

We are pleased to inform you that your manuscript is accepted for publication and is now being sent to our publisher to be included in the next available issue of EMBO Molecular Medicine.

EMBO Press Author Checklist

Corresponding Author Name: Ramon C Sun and Matthew S Gentry	
Journal Submitted to: EMBO Molecular Medicine	
Manuscript Number: EMM-2022-16029-V2	

USEFUL LINKS FOR COMPLETING THIS FORM
The EMBO Journal - Author Guidelines
EMBO Reports - Author Guidelines
Molecular Systems Biology - Author Guidelines
EMBO Molecular Medicine - Author Guidelines

Reporting Checklist for Life Science Articles (updated January 2022)
This checklist is adapted from Materials Design Analysis Reporting (MDAR) Checklist for Authors. MDAR establishes a minimum set of requirements in transparent reporting in the life sciences (see Statement of Task: 10.31222/osf.io/9sm4x). Please follow the journal's guidelines in preparing your manuscript.
Please note that a copy of this checklist will be published alongside your article.

Abridged guidelines for figures
1. Data
The data shown in figures should satisfy the following conditions:
- the data were obtained and processed according to the field's best practice and are presented to reflect the results of the experiments in an accurate and unbiased manner.
- ideally, figure panels should include only measurements that are directly comparable to each other and obtained with the same assay.
- plots include clearly labeled error bars for independent experiments and sample sizes. Unless justified, error bars should not be shown for technical replicates.
- if n<5, the individual data points from each experiment should be plotted. Any statistical test employed should be justified.
- Source Data should be included to report the data underlying figures according to the guidelines set out in the authorship guidelines on Data Presentation.

2. Captions
Each figure caption should contain the following information, for each panel where they are relevant:
- a specification of the experimental system investigated (eg cell line, species name).
- the assay(s) and method(s) used to carry out the reported observations and measurements.
- an explicit mention of the biological and chemical entity(ies) that are being measured.
- an explicit mention of the biological and chemical entity(ies) that are altered/varied/perturbed in a controlled manner.
- the exact sample size (n) for each experimental group/condition, given as a number, not a range;
- a description of the sample collection allowing the reader to understand whether the samples represent technical or biological replicates (including how many animals, litters, cultures, etc.).
- a statement of how many times the experiment shown was independently replicated in the laboratory.
- definitions of statistical methods and measures:
  - common tests, such as t-test (please specify whether paired vs. unpaired), simple χ2 tests, Wilcoxon and Mann-Whitney tests, can be unambiguously identified by name only, but more complex techniques should be described in the methods section;
  - are tests one-sided or two-sided?
  - are there adjustments for multiple comparisons?
  - exact statistical test results, e.g., P values = x but not P values < x;
  - definition of 'center values' as median or average;
  - definition of error bars as s.d. or s.e.m.

Please complete ALL of the questions below.
Select "Not Applicable" only when the requested information is not relevant for your study.

Materials

Newly Created Materials	Information included in the manuscript?	In which section is the information available? (Reagents and Tools Table, Materials and Methods, Figures, Data Availability Section)
New materials and reagents need to be available; do any restrictions apply?	Not Applicable	Not Applicable

Antibodies	Information included in the manuscript?	In which section is the information available? (Reagents and Tools Table, Materials and Methods, Figures, Data Availability Section)
For **antibodies** provide the following information:<br>- Commercial antibodies: RRID (if possible) or supplier name, catalogue number and or/clone number<br>- Non-commercial: RRID or citation	Yes	Materials and Methods

DNA and RNA sequences	Information included in the manuscript?	In which section is the information available? (Reagents and Tools Table, Materials and Methods, Figures, Data Availability Section)
**Short novel DNA or RNA including primers, probes**: provide the sequences.	Not Applicable	

Cell materials	Information included in the manuscript?	In which section is the information available? (Reagents and Tools Table, Materials and Methods, Figures, Data Availability Section)
**Cell lines:** Provide species information, strain. Provide accession number in repository **OR** supplier name, catalog number, clone number, and/**OR** RRID.	Yes	Materials and Methods
**Primary cultures:** Provide species, strain, sex of origin, genetic modification status.	Not Applicable	
Report if the cell lines were recently **authenticated** (e.g., by STR profiling) and tested for mycoplasma contamination.	Yes	Materials and Methods

Experimental animals	Information included in the manuscript?	In which section is the information available? (Reagents and Tools Table, Materials and Methods, Figures, Data Availability Section)
**Laboratory animals or Model organisms:** Provide species, strain, sex, age, genetic modification status. Provide accession number in repository **OR** supplier name, catalog number, clone number, **OR** RRID.	Yes	Materials and Methods
**Animal observed in or captured from the field:** Provide species, sex, and age where possible.	Not Applicable	
Please detail **housing and husbandry conditions**.	Yes	Materials and Methods

Plants and microbes	Information included in the manuscript?	In which section is the information available? (Reagents and Tools Table, Materials and Methods, Figures, Data Availability Section)
**Plants:** provide species and strain, ecotype and cultivar where relevant, unique accession number if available, and source (including location for collected wild specimens).	Not Applicable	
**Microbes:** provide species and strain, unique accession number if available, and source.	Not Applicable	

Human research participants	Information included in the manuscript?	In which section is the information available? (Reagents and Tools Table, Materials and Methods, Figures, Data Availability Section)
If collected and within the bounds of privacy constraints report on age, sex and gender or ethnicity for all study participants.	Not Applicable	

Core facilities	Information included in the manuscript?	In which section is the information available? (Reagents and Tools Table, Materials and Methods, Figures, Data Availability Section)
If your work benefited from core facilities, was their service mentioned in the acknowledgments section?	Yes	Acknowledgements

Design

Study protocol	Information included in the manuscript?	In which section is the information available? (Reagents and Tools Table, Materials and Methods, Figures, Data Availability Section)
If study protocol has been **pre-registered, provide DOI in the manuscript**. For clinical trials, provide the trial registration number **OR** cite DOI.	Not Applicable	
Report the **clinical trial registration number** (at ClinicalTrials.gov or equivalent), where applicable.	Not Applicable	

Laboratory protocol	Information included in the manuscript?	In which section is the information available? (Reagents and Tools Table, Materials and Methods, Figures, Data Availability Section)
Provide DOI OR other citation details if **external detailed step-by-step protocols** are available.	Not Applicable	

Experimental study design and statistics	Information included in the manuscript?	In which section is the information available? (Reagents and Tools Table, Materials and Methods, Figures, Data Availability Section)
Include a statement about **sample size** estimate even if no statistical methods were used.	Not Applicable	
Were any steps taken to minimize the effects of subjective bias when allocating animals/samples to treatment (e.g. **randomization procedure**)? If yes, have they been described?	Not Applicable	
Include a statement about **blinding** even if no blinding was done.	Not Applicable	
Describe **inclusion/exclusion criteria** if samples or animals were excluded from the analysis. Were the criteria pre-established?<br><br>If sample or data points were omitted from analysis, report if this was due to attrition or intentional exclusion and provide justification.	Not Applicable	
For every figure, are **statistical tests** justified as appropriate? Do the data meet the assumptions of the tests (e.g., normal distribution)? Describe any methods used to assess it. Is there an estimate of variation within each group of data? Is the variance similar between the groups that are being statistically compared?	Yes	Materials and Methods, Figure Captions

Sample definition and in-laboratory replication	Information included in the manuscript?	In which section is the information available? (Reagents and Tools Table, Materials and Methods, Figures, Data Availability Section)
In the figure legends: state number of times the experiment was **replicated** in laboratory.	Yes	
In the figure legends: define whether data describe **technical or biological replicates**.	Yes	

Ethics

Ethics	Information included in the manuscript?	In which section is the information available? (Reagents and Tools Table, Materials and Methods, Figures, Data Availability Section)
Studies involving **human participants**: State details of **authority granting ethics approval** (IRB or equivalent committee(s), provide reference number for approval.	Not Applicable	
Studies involving **human participants**: Include a statement confirming that **informed consent** was obtained from all subjects and that the experiments conformed to the principles set out in the WMA Declaration of Helsinki and the Department of Health and Human Services Belmont Report.	Yes	Materials and Methods
Studies involving **human participants:** For publication of **patient photos**, include a statement confirming that consent to publish was obtained.	Not Applicable	
Studies involving experimental **animals**: State details of **authority granting ethics approval** (IRB or equivalent committee(s), provide reference number for approval. Include a statement of compliance with ethical regulations.	Yes	Materials and Methods
Studies involving **specimen and field samples:** State if relevant **permits** obtained, provide details of authority approving study; if none were required, explain why.	Not Applicable	

Dual Use Research of Concern (DURC)	Information included in the manuscript?	In which section is the information available? (Reagents and Tools Table, Materials and Methods, Figures, Data Availability Section)
Could your study fall under dual use research restrictions? Please check biosecurity documents and list of **select agents and toxins** (CDC): https://www.selectagents.gov/sat/list.htm	Not Applicable	
If you used a select agent, is the security level of the lab appropriate and reported in the manuscript?	Not Applicable	
If a study is subject to dual use research of concern regulations, is the name of the **authority granting approval and reference number** for the regulatory approval provided in the manuscript?	Not Applicable	

Reporting

The MDAR framework recommends adoption of discipline-specific guidelines, established and endorsed through community initiatives. Journals have their own policy about requiring specific guidelines and recommendations to complement MDAR.

Adherence to community standards	Information included in the manuscript?	In which section is the information available? (Reagents and Tools Table, Materials and Methods, Figures, Data Availability Section)
State if relevant guidelines or checklists (e.g., **ICMJE, MIBBI, ARRIVE, PRISMA**) have been followed or provided.	Not Applicable	
For **tumor marker prognostic studies,** we recommend that you follow the **REMARK** reporting guidelines (see link list at top right). See author guidelines, under 'Reporting Guidelines'. Please confirm you have followed these guidelines.	Not Applicable	
For **phase II and III randomized controlled trials**, please refer to the **CONSORT** flow diagram (see link list at top right) and submit the CONSORT checklist (see link list at top right) with your submission. See author guidelines, under 'Reporting Guidelines'. Please confirm you have submitted this list.	Not Applicable	

Data Availability

Data availability	Information included in the manuscript?	In which section is the information available? (Reagents and Tools Table, Materials and Methods, Figures, Data Availability Section)
Have **primary datasets** been deposited according to the journal's guidelines (see 'Data Deposition' section) and the respective accession numbers provided in the Data Availability Section?	Not Applicable	
Were **human clinical and genomic datasets** deposited in a public access-controlled repository in accordance to ethical obligations to the patients and to the applicable consent agreement?	Not Applicable	
Are **computational models** that are central and integral to a study available without restrictions in a machine-readable form? Were the relevant accession numbers or links provided?	Not Applicable	
If publicly available data were reused, provide the respective **data citations in the reference list**.	Not Applicable	
